# A localized sanitation status index as a proxy for fecal contamination in urban Maputo, Mozambique

**DOI:** 10.1371/journal.pone.0224333

**Published:** 2019-10-25

**Authors:** Drew Capone, Zaida Adriano, David Berendes, Oliver Cumming, Robert Dreibelbis, David A. Holcomb, Jackie Knee, Ian Ross, Joe Brown

**Affiliations:** 1 Civil and Environmental Engineering, Georgia Institute of Technology, Atlanta, Georgia, United States of America; 2 WE Consult, Maputo, Mozambique; 3 Departamento de Geografia, Universidade Eduardo Mondlane, Maputo, Mozambique; 4 Waterborne Disease Prevention Branch, Division of Foodborne, Waterborne, and Environmental Diseases, National Center for Emerging Zoonotic and Infectious Diseases, Centers for Disease Control and Prevention, Atlanta, Georgia, United States of America; 5 Department of Disease Control, London School of Hygiene and Tropical Medicine, London, United Kingdom; 6 Department of Environmental Sciences and Engineering, University of North Carolina at Chapel Hill, Chapel Hill, North Carolina, United States of America; Purdue University, UNITED STATES

## Abstract

Sanitary surveys are used in low- and middle-income countries to assess water, sanitation, and hygiene conditions, but have rarely been compared with direct measures of environmental fecal contamination. We conducted a cross-sectional assessment of sanitary conditions and *E*. *coli* counts in soils and on surfaces of compounds (household clusters) in low-income neighborhoods of Maputo, Mozambique. We adapted the World Bank’s Urban Sanitation Status Index to implement a sanitary survey tool specifically for compounds: a Localized Sanitation Status Index (LSSI) ranging from zero (poor sanitary conditions) to one (better sanitary conditions) calculated from 20 variables that characterized local sanitary conditions. We measured the variation in the LSSI with *E*. *coli* counts in soil (nine locations/compound) and surface swabs (seven locations/compound) in 80 compounds to assess reliability. Multivariable regression indicated that a ten-percentage point increase in LSSI was associated with 0.05 (95% CI: 0.00, 0.11) log_10_ fewer *E*. *coli*/dry gram in courtyard soil. Overall, the LSSI may be associated with fecal contamination in compound soil; however, the differences detected may not be meaningful in terms of public health hazards.

## Introduction

Disparities in sanitation coverage exist across the globe: in North America and Europe 97% of the population have access to at least basic sanitation compared to 28% in Sub-Saharan Africa [1]. The United Nations Joint Monitoring Programme’s (JMP) Sustainable Development Goal (SDG) 6 calls for universal access to safely managed sanitation by 2030, which it defines as “the use of improved facilities and where excreta are safely disposed of *in situ* or treated off-site” [1]. One step below safely managed on the JMP sanitation ladder is the basic sanitation service level, defined as “use of unshared improved facilities.”

Safely managed sanitation is one of multiple water, sanitation, and hygiene (WASH) interventions designed to serve as a primary barrier to environmental enteric pathogen transmission and, subsequently, reduce enteric infections [2]. Human excreta is more likely to spread infection via multiple interacting pathways when safely managed sanitation is absent [3]. There is increasing interest in soil as an important environmental transmission pathway for enteric pathogens, especially among children who may mouth contaminated hands or objects, or directly ingest soil [4–8]. The pathways through which human excreta spreads to the environment further suggests that soil serves as a sink for enteric pathogens [3,9]. As such, the levels of fecal contamination in soils—and on other household surfaces frequently contacted by children—may provide a useful metric for assessing the fecal waste-related hazards present generally at local household and near-household scales.

Recent large health impact trials found mixed effects of WASH interventions on children’s growth and diarrhea [10–12]. Fecal-oral pathogens are transmitted through multiple pathways[2,13,14] and recent large health impact trials may have insufficiently reduced the dose of pathogens ingested by children or failed to reduce a sufficient number of transmission pathways to observe a health impact. Given that children’s growth and diarrhea prevalence are distal effects of sanitation, presumably mediated by reductions in fecal contamination, understanding and reducing fecal contamination in soil [15–17]—and other environmental matrices [18,19]—may be useful before further expensive health impact trials are conducted. Without changes to other indicators of sanitary quality (e.g. drainage, solid waste management, fecal sludge management, presence of animals, latrine flooding), simple WASH improvements (e.g. providing latrines with only a slab) may be insufficient to reduce exposure risks to fecal-oral pathogens. Reducing environmental fecal contamination may require systems-based approaches [20], including holistic, transformative interventions that ensure effective sequestration of human and animal fecal wastes both at the household and downstream in the sanitation chain.

Sanitary surveys are a systems-based approach to assess the disposal chain of human excreta and sanitary conditions [21–24]. Many existing sanitary survey instruments are intended to support the development of sanitation master plans or to identify areas in need of sanitation interventions, particularly at neighborhood or city-wide levels [21–25]. A localized (i.e., near-household) sanitary survey may be useful as a proxy for environmental fecal contamination. However, there is limited evidence of the validity of localized sanitary survey instruments as useful and reliable indicators of compound environmental fecal contamination [26].

A major challenge in evaluating environmental fecal contamination with such sanitary survey metrics is the choice of indicator organism or pathogen for reasons of cost and capacity. Statistically representative, quantitative measures of enteric pathogens or pathogen/fecal indicators in all environmental media of interest in a given setting are both time-consuming and generally prohibitively expensive [27]. Proxy measures of fecal contamination are often useful in approximating sanitary risks and evaluating sanitation status [27]. By comparing sanitary survey scores to the occurrence of *E*. *coli*, a widely used fecal indicator, in soils and on surfaces, we can evaluate the suitability of such an approach for approximating localized fecal contamination.

The objectives of our study were to (1) design and implement a sanitary survey that systematically quantified the sanitary conditions at compounds enrolled in a sanitation trial in low-income urban communities of Maputo, Mozambique; (2) evaluate whether and how the sanitary survey were associated with localized fecal hazards, as indicated by *E*. *coli* occurrence in soil and on surfaces from study compounds; and (3) identify other key variables associated with *E*. *coli* counts in courtyard soils and on surfaces in this setting. Results of this study could inform future sanitary survey validation in other settings.

## Materials and methods

### The Maputo sanitation (MapSan) trial

The Maputo metropolitan area contains 2.7 million people [28], of which about only 136,000 (5%) are served by a sewer system that is insufficiently funded for adequate maintenance [29]. Among those without sewerage, about 36% use pit latrines and 64% use pour-flush toilets leading to a pit or septic tank [25]. About 14% of on-site sanitation facilities in Maputo are shared by two or more households [30].

The MapSan Trial was a controlled, before-and-after trial to estimate the health impacts of an urban sanitation intervention [31]. The intervention consisted of private pour-flush latrines (to septic tank) shared by multiple households in compounds (S1 Fig), which were installed from 2015–2017. Areas of Maputo with a high-water table were excluded from receiving the intervention. Controls used existing shared private latrines throughout the trial. The study area was in densely populated, low-income, unplanned neighborhoods of urban Maputo, Mozambique. The study area is characterized by poor sanitary and environmental conditions, which contribute to a high burden of enteric disease and child mortality [32–35]. As a purposive, nested sub-study, this study included a selection of both intervention and control compounds enrolled in the MapSan trial.

### The localized sanitation status index

We conducted a literature review to identify methodologies to consider for adaptation that yielded six recent sanitary surveys [21–25,36]. These surveys relied on similar inputs: socioeconomic variables [21], habitation characteristics [21,24], water access and availability [21–25], the full disposal chain of human excreta [21–25], solid waste disposal methods [21–25], drainage and waste water conditions [21,23–25], latrine sharing [21,23,25], latrine hygienic conditions [21,23,25], the safety of the latrine superstructure [25], and open defecation practices (S1 Table) [36]. Of these surveys, we chose to adapt the World Bank’s Urban Sanitation Status Index (USSI) because 1) its methodology was the most feasible, and 2) it was locally relevant, as it was developed in Maputo [37].

The USSI was constructed using the guidelines proposed by the Organization for Economic Cooperation for the construction of composite indicators [38]. The USSI was developed based on the theoretical framework proposed by the World Bank’s Water and Sanitation Program (WSP), which accounts for the three main steps in on-site sanitation management: containment, emptying and transport, and treatment and disposal [39,40]. WSP recognized sanitation as a series of interlinked services and therefore included “complementary services” as the fourth component to evaluate sanitation status.[25] For each of the four components, WSP conducted a literature review to select the USSI’s indicators using the following criteria: (1) appropriate to the study context; (2) data could be easily collected; (3) sensitive to spatial or temporal change; (4) easy to interpret; (5) policy-relevant or actionable [25].

The USSI uses surveys of households and local sanitation experts to calculate nine indicators of sanitary conditions and the overall sanitary score [25,37]. In constructing the Localized Sanitation Status Index (LSSI), we retained 18 of the 20 variables from the USSI. We did not add any additional variables but did split the *transport safety* (to separate the household and community inputs present in the USSI variable) and *onsite sanitation superstructure* (the USSI used *roof* and *walls* as unique sub-variables in the on-site sanitation superstructure variable, we reported them as two variables for transparency) variables into two variables for each, for a total of 20 unique variables. We excluded the *level of treatment of excreta* variable used in the USSI from the LSSI to avoid including homogenous inputs (there was only one poorly maintained treatment plant in Maputo at the time of survey). Similarly, we excluded the *drainage canals* variable because minimal drainage infrastructure served the study area at the time of survey.

Our adaptation followed the same framework as the USSI, except the outcome of USSI was an average community level sanitation score while we chose to analyze and retain individual household data to produce a localized sanitation score. We designed household and community block leader survey questions to correspond to the 20 input variables of sanitary conditions for the LSSI (Table 1).

**Table 1:** LSSI/USSI variables.

Component	Indicator	Indicator Weight	Variable	Data Source	Variable Weight	References
**Containment**	Access to Infrastructure	14.9%	Type of on-site sanitation system	Household survey	0.7	[1,3,41,42]
			On-site sanitation sharing	Household survey	0.3	[43,44]
	Containment Safety	8.6%	Structural stability of the facility	Household survey	0.25	[41,45]
			Type of lining	Household survey	0.25	[3,45,46]
			On-site sanitation system roof	Household survey	0.125	[3,45,47]
			On-site sanitation system walls	Household survey	0.125	[3,45,47]
			Containment effectiveness	Household survey	0.25	[3,9,24]
			Groundwater level	Community block leader survey	*	[3,9,45,46]
	Hygiene	12.9%	Hygienic condition of the on-site sanitation system	Household survey	0.4	[21,23,42]
			Soap and water nearby for handwashing	Household survey	0.3	[3,43,48]
			Type of lid covering the drop hole	Household survey	0.3	[3,9,47]
**Emptying and Transport**	Access to emptying services	18.0%	Intended type of equipment to empty the latrine or septic tank	Household survey	1	[9,25,49,50]
	Transport safety	7.9%	Local amount of fecal waste transported to WWTP	Household survey	0.5	[9,25,49,50]
			Neighborhood amount of fecal waste transported to WWTP	Community block leader survey	0.5	[9,25,49,50]
**Final Disposal**	Final disposal	14.4%	Quality of disposal management	Household survey	1	[3,9,49]
			Groundwater level	Community block leader survey	*	[3,9,45,46]
**Complementary services**	Access to water supply	7.7%	Water availability for flushing and cleaning	Household survey	1	[3,23,51]
	Solid Waste Management	7.0%	Local accumulation of solid waste	Household survey	0.8	[21,25,45]
			Neighborhood accumulation of solid waste	Community block leader survey	0.2	[21,25,45]
	Storm- and greywater management	8.4%	Local accumulation of storm water	Household survey	0.5	[25,45,52]
			In-house greywater management	Household survey	0.5	[25,52,53]

*Groundwater level had no weight. It was used as a multiplier and is explained in the supporting information (S1 Text).

We assigned ordinal values ranging from 0 to 1 (in order of poorest to best sanitary conditions) to each survey response for each of the 20 input variables. Intermediate values were split evenly across the range (e.g. ordinal responses of A, B, C, and D were assigned 0, 0.33, 0.67, and 1, respectively). We weighted the 20 input variables according to the previously-implemented USSI in Maputo [37] and used weighted values created by the World Bank for Maputo to calculate the nine indicators of local sanitary conditions (Table 1). The weights for Maputo were created using the Analytic Hierarchy Process technique [54] to estimate the relative importance of each indicator from a questionnaire of 20 local sanitation experts [25,37]. Local sanitation experts included utility and local government sanitation managers, environmental health officers, NGOs and aid workers, researchers, and provincial/national government personnel. We aggregated the nine indicators according to their weight to calculate the LSSI for each compound (Table 1). We provide further detail on variable and indicator aggregation in the supporting information (S1 Text).

Recognizing that the development of the within-variable categorial weights, variable weights and indicator weights may have been subjective, we developed a simplified LSSI alternative, the Unweighted LSSI, to compare against the LSSI. We calculated the Unweighted LSSI by a simple average of the 20 LSSI variables.

### Survey groups

This survey took place from December 2017 to July 2018 (S2 Text). We trained enumerators to conduct interviews with household residents through a two-day facilitated workshop and during one week of survey piloting in December 2017, and an additional two days of survey piloting in April 2018. We trained enumerators to conduct interviews with community block leaders through a one-day facilitated workshop and one day of survey piloting in May 2018. Enumerators conducted interviews with household residents from April–July 2018 and with community block leaders in June 2018.

All questionnaires were communicated by the enumerators in either Portuguese or the local language, Changana, as requested by the respondent. Our sampling frame included one household respondent from each compound enrolled in the MapSan trial that had completed the 12-month follow-up household survey.[31] We recognized that MapSan respondents were a relatively homogenous group (women with young children). Therefore, we aimed to survey a second non-MapSan household respondent from each compound, who we identified as an adult resident of the third household on the right of the compound entrance.

In ArcGIS (ESRI, Redlands, CA) we laid a grid of 40 points across the MapSan trial area approximately 300 meters apart and determined the community block each point was located in. Enumerators visited the corresponding community block leaders and surveyed them at their homes. Community block leaders are volunteers who serve as the lowest level government officials in Maputo, and their responsibilities include mobilizing residents to look after public infrastructure and cleanliness [25]. We matched household survey responses to the nearest community block leader by GPS location for neighborhood-level LSSI inputs (S2 Fig).

### Environmental sampling site selection

We calculated preliminary LSSI scores to identify compounds for environmental sampling by applying the LSSI methodology to household survey data collected during the most recent (24-month) follow-up visits of the MapSan trial. In calculating the preliminary LSSI, we ignored neighborhood and certain household-level variables that were not collected as part of the MapSan survey conducted from 2017–2018. Based on resource constraints we aimed *a priori* to sample from 80 total compounds: those with the 40 highest and 40 lowest scores on the preliminary LSSI to test the hypothesis that the LSSI varies with objective measures of fecal contamination. The selection of compounds at the extremes of LSSI equipped the study with the greatest power to detect differences in environmental fecal contamination between relatively low and high LSSI scores. We conducted environmental sampling of soils and surfaces from May–June 2018.

### Soil sampling

At each compound we collected nine soil samples at the following locations, as identified by an adult member of a household enrolled in the MapSan trial: 1) the most frequently used compound entrance; 2) the household entrance, 3) the latrine entrance; 4) the food preparation area; 5) the dish-washing area; 6) clothes washing area; and 7) the area solid waste was stored; 8) the center of the compound yard we estimated by approximating the midpoint of all the household entrances in a compound; and 9) a second household entrance, from a household not enrolled in the MapSan study, selected by locating a household entrance across the compound yard from the first household entrance. If there was no household across the compound yard from the first household (sample location 9), we selected the household entrance that was farthest away from the first household entrance. We collected all soil samples using a metal scoop that was disinfected with 10% bleach and 70% ethanol between uses. For each sample, we used the metal scoop to homogenize a 10 cm x 10 cm x 1 cm volume of soil, which we transferred into one 5-mL cryotube and three 2-mL cryotubes. Soil samples remained on ice packs after collection and were processed within 6 hours of collection. A soil sample was recorded as “moist” or “dry” based on whether it was visibly wet at the time of collection (S3 Text). Using an estimate of the sun’s trajectory from approximately 9:00 am to 3:00pm on the day sampling took place (sampling took place during these hours each day) and the presence of nearby coverings (e.g. trees and houses), we estimated daily sun exposure, classifying each sample as “shaded”, “partially shaded” or in “direct sunlight” (S3 Text).

Bacteria were eluted from soil using modified methods from Boehm et al. [55], similar to methods reported elsewhere [56–58]. Briefly, we eluted approximately one gram of soil in 100 mL of distilled water using a 532-mL self-standing Whirl-Pak bag (Nasco, Fort Atkinson, WI). We manually shook soil samples for two minutes and then allowed samples to settle for 15 minutes. We aliquoted one mL of supernatant onto Compact Dry plates for quantification of *E*. *coli* (Compact Dry^TM^ EC, VWR, Vienna, Austria). We incubated the Compact Dry plates at 37°C for 24 hours as per the manufacturer’s instructions. We processed a separate one-gram soil sample from the same cryotube for replicate analysis of each sample and ran a negative control for every 9 soil samples. When one or both replicate samples yielded colonies too numerous to count, we tested a third sample from the same cryotube using a 1:15 dilution of the supernatant. We measured moisture content of soil samples using the microwave oven method [4,58,59]. We calculated *E*. *coli* counts in colony forming units (CFUs) per gram dry soil by a simple average of the two replicate values. Based off the manufacturer’s instructions and the dilutions used, the lower limit of detection was 2 log_10_ CFU *E*. *coli* per gram of soil, not accounting for moisture content, and the upper limit of detection was 6.48 log_10_ CFU *E*. *coli* per gram of soil.

### Swab sampling

At each compound we collected fourteen swab samples at seven locations that were identified by an adult in a household enrolled in the MapSan trial. The household member indicated or provided: 1) the most frequently used compound entrance door or door frame, 2) the household entrance door, 3) latrine entrance door or door frame, 4) a food preparation surface, 5) a plate used to serve food, 6) a plastic chair (we swabbed the horizontal seat surface), and 7) the most frequent play toy of a child from the subject’s household. We recorded whether each surface was visibly dirty at the time of sampling. We swabbed adjacent surface areas of 100 cm^2^ and 10cm^2^ using a method adapted from Hedin et al. and similar to other studies [4,60]. We swabbed each surface with two sterile nylon flocked swabs (Copan Diagnostics, Murrieta, CA). First, we wetted a swab with sterile ¼ strength Ringer’s solution (MilliporeSigma, Burlington, MA) and swabbed the entire surface in the horizontal, vertical and diagonal directions. Then we repeated this process on the same surface using a dry swab. We cut the swab end of the wet and dry swabs using scissors sterilized with 10% bleach and 70% ethanol and inserted the swabs into an Ojal Test Kit (Ojal Water Technologies Pvt. Ltd, Bangalore, India, www.ojalwatertest.com), an *E*. *coli* test that uses Aquatest medium [61,62] to produces a color change in the presence of *E*. *coli* (S4 Text). We added either 100 mL or 10 mL of distilled water to the Ojal test kits with the swabs in them, according to the manufacturer’s instructions, and then shook samples for two minutes to elute *E*. *coli* from the swabs. The limit of detection from this test was ≥1 *E*. *coli* per 10 cm^2^ and ≥1 *E*. *coli* per 100cm^2^. We ran a blank control of only distilled water and a second control containing distilled water and a swab wetted in ¼ strength Ringer’s solution for every seven samples processed. We incubated the Ojal Test kits at 37°C for 24 hours, per the manufacturer’s instructions, before reading.

### Data analysis

We analyzed data in R version 3.5.0 (R Foundation for Statistical Computing, Vienna, Austria). To account for nested clusters of households within clusters of compounds we used linear mixed-effect models (LMM) on log_10_-transformed values of CFU *E*. *coli* per dry gram of soil to perform linear regression modelling, and generalized linear mixed-effect models (GLMM) on binary detect/non-detect *E*. *coli* in soil, and binary detect/non-detect *E*. *coli* on surfaces to perform Poisson regression modelling. In our models, *E*. *coli* concentration or detect/non-detect was our dependent variable and the LSSI was our independent variable. We used the “lme4” package in R for regression analysis and used a Poisson (log) distribution for calculation of unadjusted risk ratios (RR) and adjusted risk ratios (aRR) [63].

We *a priori* decided to adjust for sunlight, location of the soil sample in the courtyard, a compound’s wealth index, and presence of chickens and ducks (S3 Fig) [64], as literature suggests these variables may be important confounders [4,57,65]. We did not adjust for soil moisture as both sunlight and the location of a soil sample in the courtyard were associated with soil moisture and moisture was already accounted for by normalizing *E*. *coli* concentrations by moisture content (per gram dry soil). *A priori* we decided to evaluate associations between *E*. *coli* in soil and the LSSI score continuously and by quartiles. Given the low levels of *E*. *coli* detected on surfaces and suggested confounders from a previous study [4], we decided to analyze the detection/non-detection of *E*. *coli* on surfaces and adjusted for visible dirt on the surface, intra-compound location, and wealth [4].

We assigned *E*. *coli* concentrations in non-detect soil samples to half the value of the LLOD [15,66] and we did not observe any samples with *E*. *coli* concentrations above the upper limit of detection. We calculated household wealth using eight of the ten inputs from the Simple Poverty Scorecard for Mozambique [67]. We excluded number of beds and latrine type from our calculation of household wealth because of limited data and latrine type due to our cross sectional design [68]. When we surveyed two households in a compound, we used the mean wealth score as the compound wealth score and the mean LSSI as the compound LSSI.

### Ethical approvals

Before conducting a survey with an adult household member or *a* community block leader we obtained written informed consent from the respondent. We obtained verbal consent from the head of a compound to perform environmental sampling and requested permission to sample from all compound heads at least one day in advance. The study protocols were approved by the Comité Nacional de Bioética para a Saúde (CNBS), Ministério da Saúde (333/CNBS/14, 81/CNBS/18), the Ethics Committee of the London School of Hygiene and Tropical Medicine (Reference # 8345) and the Institutional Review Board of the Georgia Institute of Technology (Protocol # H15160, # H18027). The associated MapSan trial has been registered at ClinicalTrials.gov (NCT02362932).

## Results

### Household characteristics

We visited 147 households at 80 MapSan compounds (13 compounds lacked a second household to interview; S2 Table) and conducted interviews with 133 households at 75 MapSan compounds (three respondents did not consent and 11 had moved away). The median amount of time respondents lived in their home was nine years and the average was 14 years (S2 Table). Compounds contained an average of four families, 17 people, two children under the age of five, and scored 33 out of 81 (Standard Deviation (SD) = 11) on the Mozambique Simple Poverty Scorecard (S2 Table) [67]. We observed human feces in the compound yard or on the floor of the on-site sanitation system at 11% (n = 9) of compounds, used children’s diapers on the ground or in a pile of garbage at 13% of compounds (n = 10), and standing water at 49% (39) compounds (S2 Table). We observed animals in 59% (n = 47) of compounds consisting of cats (n = 32, [40%]), chickens (n = 12, [15%]), ducks (n = 8, [10%]), dogs (n = 7, [9%]), and pigeons (n = 1, [1%]). The on-site sanitation systems at the 80 environmental sampling compounds were predominantly pour-flush to pit or septic tank (n = 50, [63%]), while 16% (n = 13) possessed pit latrine with concrete slab, and 21% (n = 17) possessed a pit latrine without a concrete slab (S2 Table). Additionally, 39 of 40 community block leaders (98%) consented to an interview.

### Soils

We collected 720 soil samples from 80 MapSan compounds and detected *E*. *coli* in 74% of samples with a mean concentration of 4.10 log_10_ CFU *E*. *coli* per gram of dry soil (standard deviation = 4.78 log_10_) and a median of 2.77 log_10_ CFU *E*. *coli* per gram of dry soil (range = no detect (ND), 6.14 log_10_). The mean difference between the replicate soil samples analyzed from each location was 3.76 log_10_ CFU *E*. *coli* per gram of dry soil, the median was 2.50 log_10_ CFU *E*. *coli*, and the Pearson’s correlation coefficient was 0.84 (S5 Text). We most frequently detected *E*. *coli* in soils from washing areas for clothes (91%) and dishes (90%), while least frequently detected *E*. *coli* in soils at the compound center (60%) and the non-MapSan household entrance (59%) (Table 2). Among intra-compound locations, the highest average *E*. *coli* concentration was found at the dishwashing area (mean 4.54 log_10_ CFU *E*. *coli*), while the center of the compound yard had the lowest concentrations (mean 3.66 log_10_ CFU *E*. *coli*). We noted 65% of samples as visibly wet at the time of sampling and 35% as visibly dry; we most frequently observed soil from the clothes washing area (85%, [n = 68/80]) and dishwashing area (90%, [n = 72/80]) as visibly wet (S3 Table). We recorded that 13% (95) of sample locations experienced complete sunlight throughout the day, 30% (288) both direct sunlight and shade, and 47% (337) remained completely shaded. We estimated sun exposure status to be similar across intra-compound locations, except for the center of the compound yard which was estimated to be in full sun (29%, [n = 23/80) more often than other locations and the food preparation area which was estimated to be complete shade (65%, [n = 52/80]) more often than the other locations (S4 Table).

**Table 2:** CFU *E*. *coli* counts at intra-compound locations.

Intra-compound location	≥LLOD	≥10^3^	≥10^4^	Mean (log_10_)	SD	Median (log_10_)	Range
Clothes Washing Area	91%	60%	20%	4.08	4.49	3.28	(ND, 5.30)
Dish Washing Area	90%	60%	26%	4.54	5.20	3.21	(ND, 6.14)
Garbage Storage Area	81%	54%	26%	4.35	4.71	3.06	(ND, 5.48)
Latrine Entrance	76%	51%	18%	3.96	4.40	3.05	(ND, 5.29)
MapSan Household Entrance	73%	36%	8%	3.74	4.35	2.42	(ND, 5.24)
Compound Entrance	69%	40%	16%	3.98	4.55	2.48	(ND, 5.46)
Food Prep Area	69%	31%	13%	3.98	4.48	2.42	(ND, 5.25)
Compound Center	60%	24%	8%	3.66	4.26	1.89	(ND, 5.14)
Non-MapSan Household Entrance	59%	33%	11%	3.73	4.18	2.32	(ND, 4.97)
All Locations	74%	43%	16%	4.10	4.78	2.77	(ND, 6.14)

### Swabs

We swabbed adjacent 100cm^2^ and 10cm^2^ surfaces at 560 locations in 80 MapSan compounds, of which 23% appeared visibly dirty. The Ojal Test yielded *E*. *coli* concentrations of ≥1 *E*. *coli* per 100cm^2^ at 3.4% of the 100cm^2^ surfaces and ≥1 *E*. *coli* per 10cm^2^ at 2.9% of the 10cm^2^ surfaces. We detected *E*. *coli* from either the 100cm^2^ or 10cm^2^ surface at 5.4% of swab locations.

### The complete LSSI

**Fig 1:**
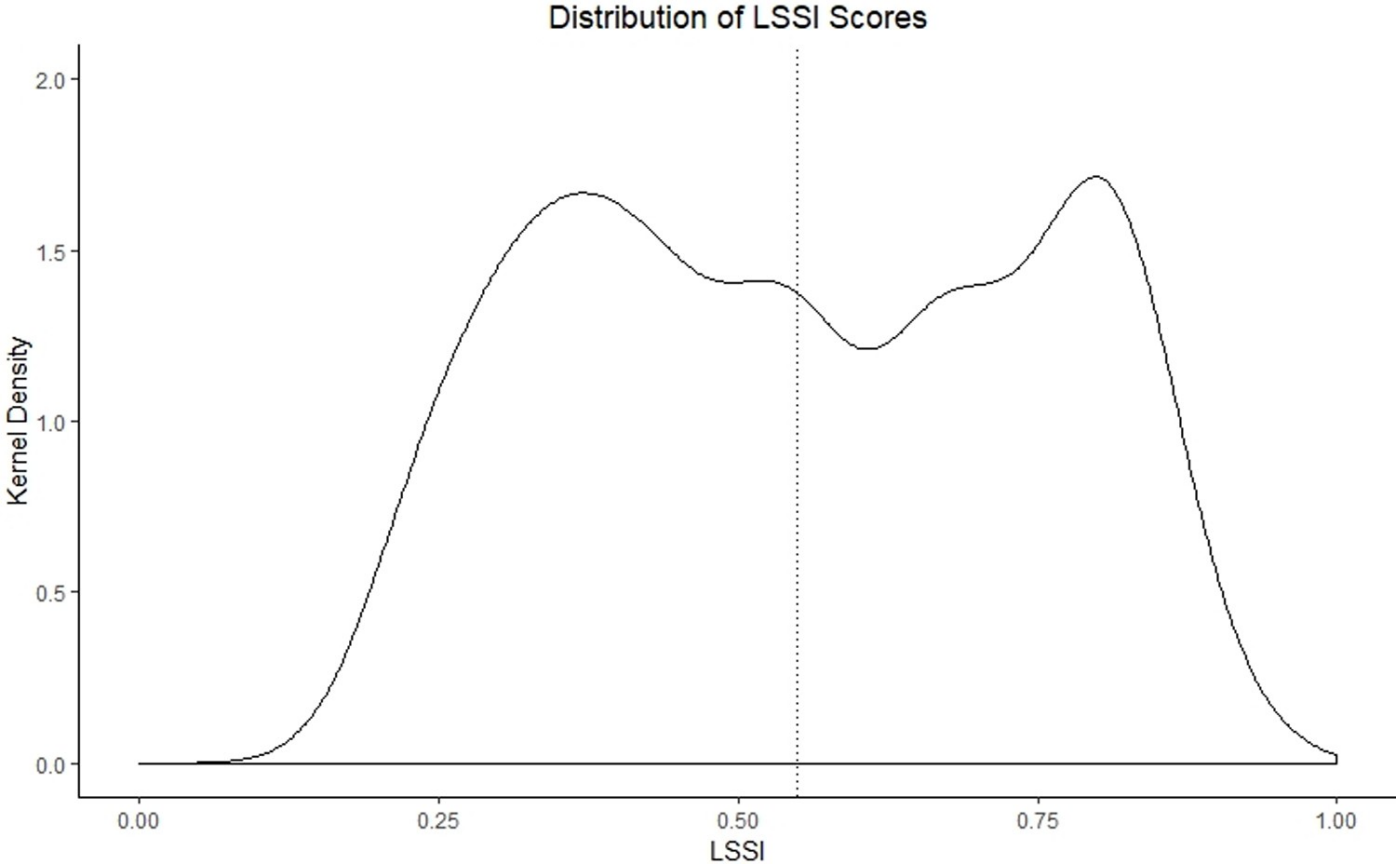
Kernel density plot of complete LSSI results.

LSSI scores ranged from 0.20 to 0.91, with a mean of 0.55 (SD = 0.20) and the distribution was bimodal (Fig 1). In the 50 compounds where we interviewed two respondents, the average intra-compound LSSI variation between respondents was 0.12 (median: 0.06).

### Continuous *E*. *coli* counts

Using multivariable regression and adjusted for sun exposure status, intra-compound location, presence of chickens and ducks, and household wealth, a ten-percentage point increase in the LSSI was associated with 0.05 log_10_ fewer CFU *E*. *coli* per gram dry soil (95% CI: -0.10, 0.00; Table 3 and S4 Fig). However, *E*. *coli* counts in soil were generally heterogenous across the range of LSSI scores (S5 Fig). Similarly, a ten-percentage point increase in the Unweighted LSSI was associated with 0.07 log_10_ fewer CFU *E*. *coli* per gram dry soil (95% CI: -0.13, -0.01). Four of the 20 LSSI variables were individually associated with log_10_-transformed *E*. *coli* counts in soil. A ten-percentage point increase in the *on-site sanitation sharing* variable was associated with 0.06 log_10_ fewer CFU *E*. *coli* per gram dry soil (95% CI: -0.10, -0.02), the *groundwater level* variable was associated with 0.03 fewer log_10_ fewer CFU *E*. *coli* per gram dry soil (95% CI: -0.06, 0.00), the *quality of disposal management* variable was associated with 0.05 log_10_ fewer CFU *E*. *coli* per gram dry soil (95% CI: -0.09, -0.01), and the *neighborhood accumulation of solid waste* variable was associated with 0.03 log_10_ fewer CFU *E*. *coli* per gram dry soil (95% CI:-0.06, 0.00) (S5 Table). We did not observe significant associations between the LSSI, when divided by quartile, and *E*. *coli* concentrations in soil. Adjusted *E*. *coli* concentrations in soil were significantly associated with shade (higher in full shade vs. full sun), moisture (higher in visibly wet vs. dry soil), and chicken presence (higher with chickens present).

**Table 3:** Uni- and multi-variable regression models for log_10_-transformed *E*. *coli* concentrations in soil and adjusted for sunlight, intra-compound location, compound wealth, chickens and ducks.

Soil Covariates	Description	Reference	Univariable β (95% CI)	Multivariable β (95% CI)
**Complete LSSI**	Localized Sanitation Status Index	Ten-percentage point increase	-0.06 (-0.13, 0.00)	-0.05 (-0.11, 0.00)
**Unweighted LSSI**	Simple average of the 20 LSSI variables		-0.09 (-0.17, -0.01)	-0.07 (-0.13, -0.00)
**LSSI: Q2**	LSSI divided into quartiles	Q1	-0.03 (-0.40, 0.34)	0.01 (-0.30, 0.31)
**LSSI: Q3**			-0.40 (-0.77, -0.03)	-0.29 (-0.60, 0.02)
**LSSI: Q4**			-0.31 (-0.68, 0.06)	-0.25 (-0.56, 0.07)
**Sunlight: partial sun**	Estimated daily sun exposure: full sun, partial sun, full shade	Full sun	0.19 (-0.04, 0.42)	0.13 (-0.10, 0.35)
**Sunlight: full shade**			0.47 (0.23, 0.71)	0.39 (0.16, 0.62)
**Moisture**	Soil sample classified as "visibly wet" or "dry"	Dry	0.97 (0.83, 1.11)	0.83 (0.69, 0.98)
**Compound entrance**	One of nine sample locations where soil was collected from each compound	Center of the compound yard	0.35 (0.08, 0.61)	0.29 (0.02, 0.56)
**MapSan household entrance**			0.22 (-0.04, 0.49)	0.15 (-0.13, 0.42)
**Non-MapSan household entrance**			0.21 (-0.05, 0.47)	0.14 (-0.13, 0.41)
**Latrine entrance**			0.58 (0.31, 0.84)	0.45 (0.18, 0.73)
**Food preparation area**			0.27 (0.00, 0.53)	0.19 (-0.08, 0.47)
**Dish washing area**			0.89 (0.63, 1.15)	0.82 (0.55, 1.10)
**Clothes washing area**			0.86 (0.60, 1.12)	0.75 (0.48, 1.02)
**Garbage storage area**			0.80 (0.54, 1.06)	0.74 (0.47, 1.01)
**Wealth Index**	Wealth quartile	1-quartile increase	-0.14 (-0.25–0.02)	-0.09 (-0.19, 0.01)
**Chickens**	Chickens present in the compound	No chickens	0.94 (0.61, 1.26)	0.66 (0.33, 0.99)
**Ducks**	Ducks present in the compound	No ducks	0.73 (0.30, 1.16)	0.42 (-0.06, 0.89)

### Any *E*. *coli* detection

Using multivariable Poisson regression and adjusted for sun exposure status, intra-compound location, presence of chickens and ducks and household wealth, we found a ten-percentage point increase in the LSSI had no apparent association with detection of *E*. *coli* (aRR: 0.98, 95% CI: 0.94, 1.02; Table 4). We did not find any apparent associations between the LSSI divided into quartiles and *E*. *coli* in soil. Additionally, visibly wet soil was associated with greater risk of detection of *E*. *coli* in soil.

**Table 4:** Logistic regression models using detect/non-detect *E*. *coli* as the response variable.

Soil Covariates	Reference	RR	aRR
**Complete LSSI**	Ten-percentage point increase	0.97 (0.93, 1.02)	0.98 (0.94, 1.02)
**Unweighted LSSI**		0.96 (0.91, 1.01)	0.97 (0.92, 1.02)
**LSSI Q2**	Quartile 1	0.91 (0.72, 1.16)	0.95 (0.73, 1.22)
**LSSI Q3**		0.87 (0.68, 1.10)	0.90 (0.70, 1.15)
**LSSI Q4**		0.82 (0.63, 1.05)	0.84 (0.65, 1.09)
**Partial sun**	Full Sun	1.24 (0.93, 1.67)	1.19 (0.88, 1.63)
**Shade**		1.30 (0.98, 1.75)	1.27 (0.94, 1.73)
**Visibly wet**	Visible Dry	1.84 (1.51, 2.26)	1.77 (1.42, 2.23)
**Food Prep Area**	Compound yard center	1.15 (0.78, 1.69)	1.12 (0.75, 1.69)
**Compound Entrance**		1.15 (0.78, 1.69)	1.14 (0.77, 1.71)
**MapSan Household Entrance**		1.21 (0.83, 1.78)	1.19 (0.80, 1.79)
**Non-MapSan Household Entrance**		0.98 (0.65, 1.47)	0.96 (0.63, 1.46)
**Latrine Entrance**		1.27 (0.87, 1.86)	1.23 (0.83, 1.83)
**Garbage Storage Area**		1.35 (0.93, 1.97)	1.32 (0.91, 1.97)
**Dish Washing Area**		1.50 (1.04, 2.17)	1.47 (1.01, 2.17)
**Clothes Washing Area**		1.52 (1.06, 2.20)	1.49 (1.04, 2.19)
**Chicken Present**	No chickens	1.32 (1.06, 1.63)	1.23 (0.96, 1.56)
**Duck Present**	No ducks	1.23 (0.94, 1.58)	1.07 (0.75, 1.49)
**Wealth Index**	1-quartile increase	0.93 (0.73, 1.09)	0.94 (0.87, 1.02)
**Compound Surface Covariates**	**Reference**	**RR**	**aRR**
**LSSI**	Ten-percentage point increase	0.97 (0.77, 1.24)	0.97 (0.75, 1.23)
**Surface visibly dirty**	Not visibly dirty	1.25 (0.47, 2.97)	0.91 (0.31, 2.40)
**Plastic chair**	Compound Entrance	1.80 (0.62, 5.86)	1.80 (0.62, 5.87)
**Food prep surface**		0.20 (0.01, 1.24)	0.20 (0.01, 1.23)
**Dinner Plate**		0.20 (0.01, 1.24)	0.20 (0.01, 1.23)
**MapSan Household door**		0.40 (0.06, 1.86)	0.40 (0.06, 1.85)
**Latrine door**		0.60 (0.12, 2.44)	0.60 (0.12, 2.45)
**Child’s toy**		0.80 (0.20, 3.02)	0.83 (0.19, 3.35)
**Wealth index**	1 quartile increase	1.00 (0.64, 1.56)	1.00 (0.64, 1.57)

Soil models adjusted for sunlight, intra-compound location, compound wealth, chickens and ducks. Surface models adjusted for visible dirt, location, and compound wealth

No covariates were significantly associated with the detection of *E*. *coli* on compound surfaces in univariable or multivariable regression after controlling for visible dirt on a surface, intra-compound location, and wealth index.

## Discussion

At compounds in low-income urban Maputo with sanitation shared by multiple households, our adapted sanitary survey methodology, the LSSI, was associated with continuous measures of *E*. *coli* from compound soils, but not with binary measures of *E*. *coli* in soils or from compound surfaces. However, we observed a modest 0.05 log_10_ CFU decrease in *E*. *coli* in compound soil per ten-percentage point increase in the LSSI, which is smaller than expected, given the range of WASH characteristics across surveyed sites. Thus, a theoretical compound with an LSSI of zero that improved its sanitary conditions to achieve an LSSI of one would experience an average reduction in *E*. *coli* concentrations of only 0.50 log_10_ per gram dry soil in this setting. These findings are consistent with a large, systematic study of environmental contamination in Bangladesh, where seemingly large changes in sanitation—e.g. the presence vs absence of a latrine—were associated with only a 0.56 log_10_ reduction of *E*. *coli* in soil [57]. Animals may also be important contributors to environmental fecal contamination in this setting. Though statistically significant, the observed reductions in *E*. *coli* concentrations are minimal and may not reflect a meaningful difference in environmental contamination, and potential subsequent risks of exposure to feces-associated enteric pathogens. *E*. *coli* in soils from this environment were widely detected (74% of samples) and in high concentrations (mean: log_10_ 4.10), so relative differences in *E*. *coli* may not reflect actual differences of public health relevance.

Our goal was to assess the potential for an association between a policy-relevant metric in use by the World Bank and by cities in Rwanda, Zambia and Mozambique with measures of fecal contamination [37]. Our results suggest that sanitary surveys may serve as useful proxies for localized environmental fecal contamination; the LSSI encompassed relevant sanitary hazards that impacted the spread of human fecal contamination into the environment, thus an association with measures of *E*. *coli* in soil was anticipated. However, the LSSI should be improved upon to attempt to produce a proxy for fecal contamination that associates with log-level reductions in environmental fecal contamination of public health significance. The association between the access to infrastructure indicator and measures of *E*. *coli* in soil was greater than association with the complete LSSI. While important for hygiene, the presence of soap and water for handwashing likely had little impact on the spread of fecal contamination into compound soil. Most households in Maputo reported never having emptied their on-site sanitation system [30]; emptying frequency is dependent on the type of on-site sanitation system and the depth of the water table such that sanitation facilities in Maputo take on average one to five years to fill up [30]. How compounds intended to empty their on-site sanitation system may not be temporally relevant to a cross-sectional sanitary survey. Future iterations of the LSSI may improve their utility by only including variables with a biologically plausible pathway to contribute to localized fecal contamination. In lieu of expert weights which may be subjective, these pathways could be weighted based on the volume, frequency, and likelihood for fecal contaminations to spread into the environment.

As in other low-income settings globally, results from our adjusted estimates indicate animals—and especially chickens—may make a significant contribution to the onsite burden of feces. In fact, recent evidence has suggested onsite fecal contribution from animals may be more than feces from humans, including in urban areas [69]. Non-human fecal contamination by domestic or wild animals can contribute to detection of fecal indicators and may indicate presence of zoonotic enteric pathogens [6]. Consistent with a cross sectional study in Bangladesh, chickens were associated with higher *E*. *coli* counts in soil compared to other animals [57]. The ubiquitous fecal contamination observed in this and other studies [15,16,57] in low-income settings may limit the ability for WASH interventions to consistently reduce environmental fecal contamination [70]. Future iterations of sanitary surveys would benefit from including the presence of animals or animal feces as inputs.

After feces is introduced to the environment, the persistence of enteric pathogens is dependent on time, temperature, soil moisture content, and exposure to UV radiation from sunlight among other factors [42]. Consistent with other studies, we found concentrations of *E*. *coli* in soil to be associated with the sun exposure status of a sample and whether the sample was visibly wet [4,57]. Despite sampling during the dry season, nearly two-thirds of soil samples were visibly wet, and we observed standing water at almost half of compounds. Unsurprisingly, we detected *E*. *coli* most frequently from locations where soil was most frequently visibly wet, the areas where water-based activities such as dishwashing and clothes washing were performed [4].

In sanitation assessments latrine entrances are typically assumed to be directly impacted by the intervention. However, among the nine intra-compound locations we tested *E*. *coli* at the latrine entrance was the third most prevalent and sixth highest in concentration. The heterogeneity of *E*. *coli* concentrations among intra-compound locations emphasizes the importance of spatial standardization for soil sampling. Soil samples should be collected from locations where similar activities are performed across sites. Our results suggest that sites such as a child’s most recent play area or where a child most recently spent time [71] may not be sufficiently standardized for soil sampling in this and similar contexts.

Swabs of common compound surfaces yielded infrequent detection of fecal contamination across surfaces in this context. We most often detected *E*. *coli* on plastic chairs, which we suspect is a result of swabbing the horizontal seat of the plastic chair which may collect dirt and debris. All entrance swab surfaces were vertical, while kitchen related surfaces are typically cleaned regularly. A similar study in Tanzania found vertical latrine wall surfaces had the lowest *E*. *coli* counts compared to other common household surfaces [4]. We did not account for how recently each surface was cleaned, which may have been heterogenous and we did not specify the type of child’s play toy or food preparation surface for swab sampling. These factors may explain limited detection of *E*. *coli* on surfaces. Exclusively swabbing horizontal surfaces such as floors [18], or identical sentinel objects such as a child’s play toy, may be better approaches to standardize swab surfaces among households [19,72,73].

*E*. *coli* in soil is an imperfect indicator of sanitation-related fecal contamination in this context and the *E*. *coli* we detected may not have come from human sources, as supported by our observed associations between chicken presence and *E*. *coli* in soil. Previous work has suggested *E*. *coli* may be indigenous to soils in the tropics [74,75]. Soil-borne *E*. *coli* can grow and replicate when incubated at 30–37°C and can persist longer than one month when temperatures exceed 25^°^C, which is common year-round in Maputo [76]. Furthermore, not all *E*. *coli* are pathogenic and *E*. *coli* do not serve as an adequate indicator for enteric pathogens in many settings [15,16,77]. Further molecular analyses of these samples will be useful to understand whether and to what extent enteric pathogens are detected in soils from these sites.

Our study has several important limitations. The sample size of 80 compounds limited the number of covariates included in models and statistical power, including multivariable assessment of variables (such as the presence of chicken or ducks) that were infrequently observed. Additionally, we did not collect data to differentiate between compounds with penned animals and free-roaming animals, which may have impacted local environmental fecal contamination. The LSSI did not include disposal of children’s feces, which, if improperly disposed of, may be spread fecal contamination into the environment [3]. The LSSI included observed human feces in and around the latrine, but open defecation rates are difficult to capture in a cross-sectional study and may vary among households in a compound [78].The pre-selection of compounds enrolled in the MapSan trial was purposive; thus our conclusions may not be generalizable to all compounds in low-income areas of Maputo, or broader contexts. The range of the LSSI in the compounds we sampled did not include many compounds with LSSI values close to 0 and 1; a larger sample size may be useful in future research to capture compounds at the extremes. The absence of association between LSSI quartiles and continuous *E*. *coli* counts may have been due to a small sample size or may suggest a non-linear relationship and could be an area of future research. LSSI weights developed from surveys of local sanitation experts may have been subjective and may not have best associated with localized fecal contamination. Substantial heterogeneity existed between sample location and sample type despite our intention to select comparable sites for soils and swab samples between compounds. Other statistical approaches may be more useful to optimize the LSSI. For example, future research could use decision tree analysis to determine which variables have the greatest impact on fecal contamination.

In low-income, pathogen- and fecal contamination-rich, urban settings where sanitary conditions are poor, our study suggests better sanitary conditions measured via a sanitary survey may be associated with lower measures of environmental fecal contamination relative to poorer scores, though the absolute difference in contamination between poor and better sanitary conditions is minor and the association we found was borderline significant. There was no significant difference in the complete LSSI’s association with concentrations and detection of *E*. *coli* in soil compared with the unweighted LSSI alternative, suggesting a need for improved variable selection and weights. Further research should explore the inclusion of animals as sanitary survey inputs and how to optimize sanitary survey weighting schemes. The LSSI provides a helpful first iteration of a proxy for environmental fecal contamination in low-income settings where analysis of environmental samples is not feasible.

## Supporting information

S1 Fig MapSan compound diagram and examples of intra-compound locations. (PDF)

S1 Table Six sanitary surveys from literature review. (PDF)

S1 Text Additional LSSI information. (PDF)

S2 Text Survey questions in Portuguese and English. (PDF)

S3 Text Descriptive definitions. (PDF)

S2 Fig Map of project area. (PDF)

S4 Text Validation of Ojal Test Kit. (PDF)

S3 Fig Directed acyclic graph for model selection. (PDF)

S4 Fig LSSI Model diagnostics. (PDF)

S2 Table Household and compound characteristics. (PDF)

S5 Text Explanation of soil replicate results. (PDF)

S3 Table Visibly wet soil by intra-compound location. (PDF)

S4 Table Soil sun exposure by intra-compound location. (PDF)

S5 Fig *E*. *coli* vs LSSI scatterplots. (PDF)

S5 Table Association of LSSI variables with *E*. *coli* counts in soil. (PDF)
