# Supplementary material for: A localized sanitation status index as a proxy for fecal contamination in urban Maputo, Mozambique
Source: PLoS One. 2019 Oct 25;14(10):e0224333. doi: 10.1371/journal.pone.0224333 (PMC6814227; doi:10.1371/journal.pone.0224333)

S1 Fig. MapSan compound diagram and examples of intra-compound locations

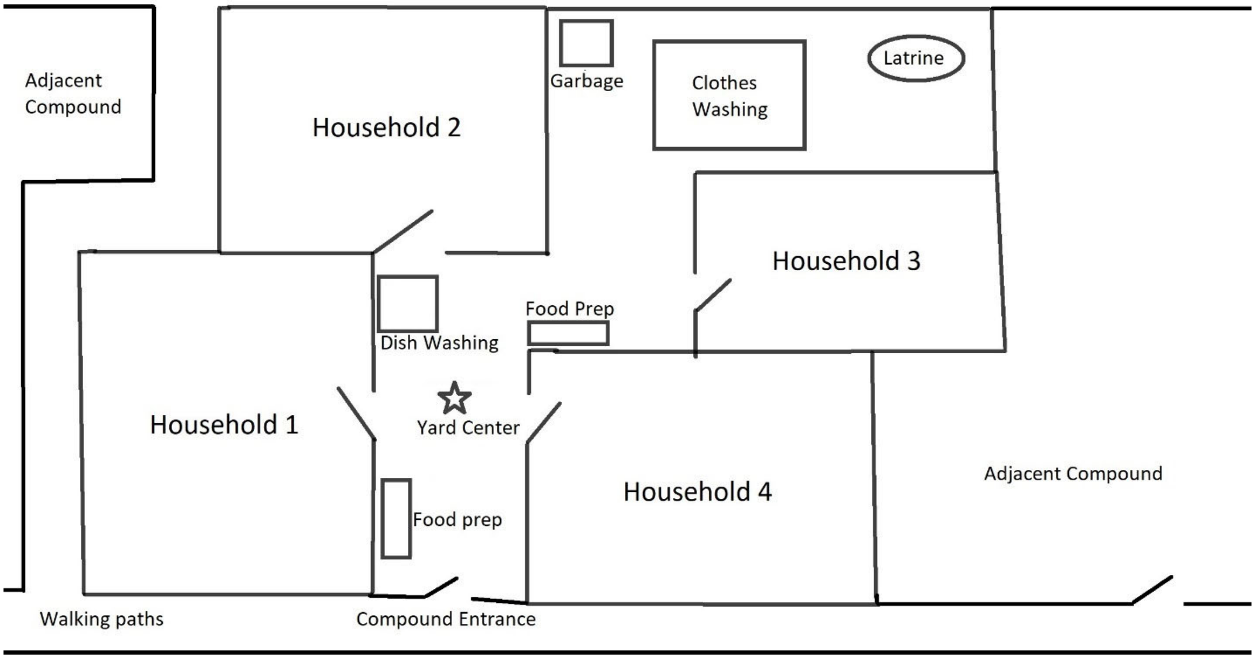

1. Example of a food preparation area:

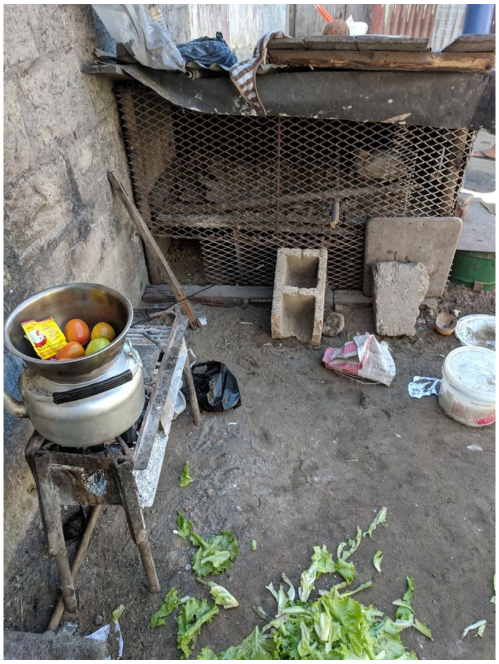

2. Example of a garbage storage area

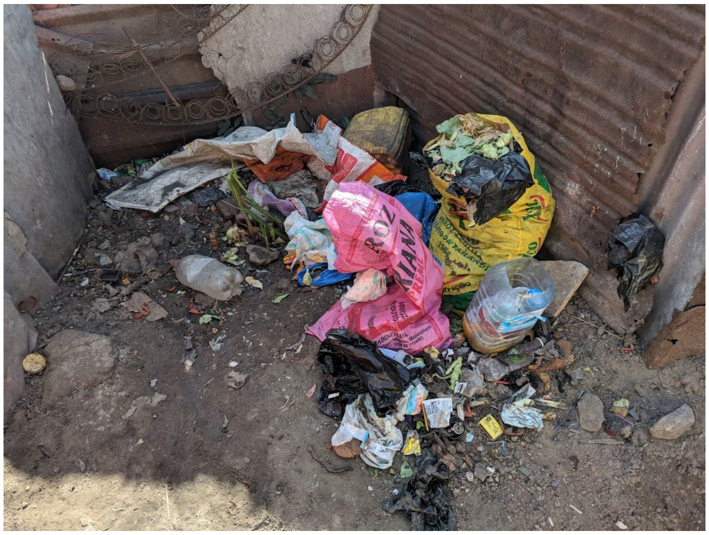

3. Example of a latrine entrance: Entrance on the left was used for bathing and the entrance on the right contained the latrine drophole.

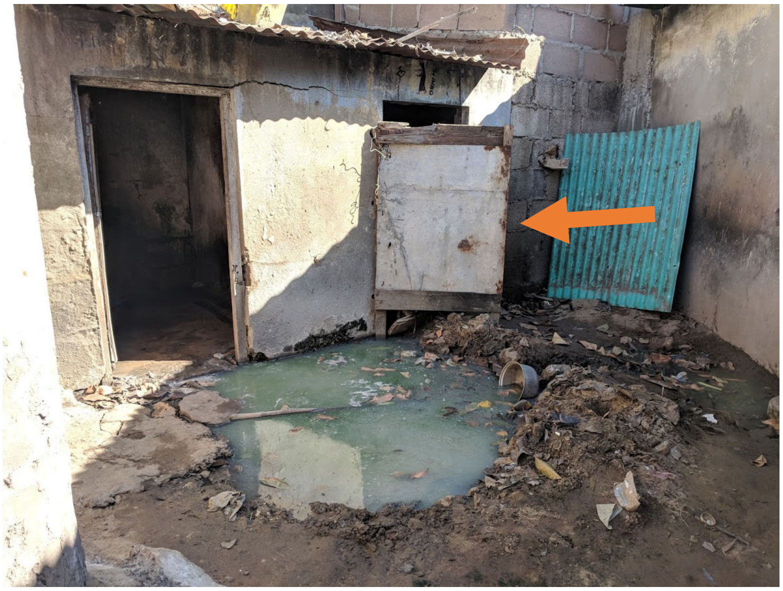

4. Example of a household entrance:

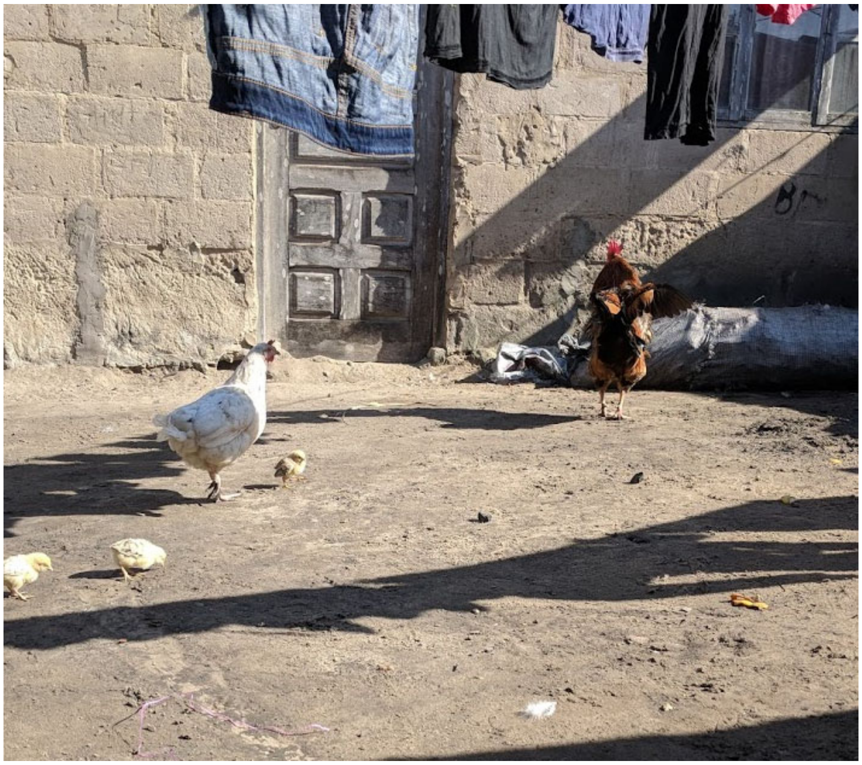

5. Example of a dish washing area:

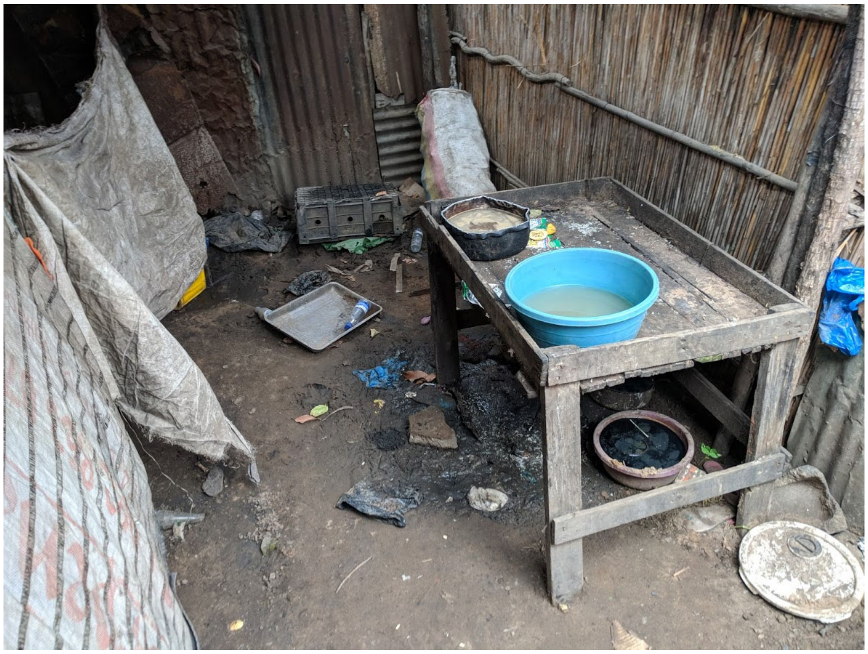

6. Example of a clothes washing area:

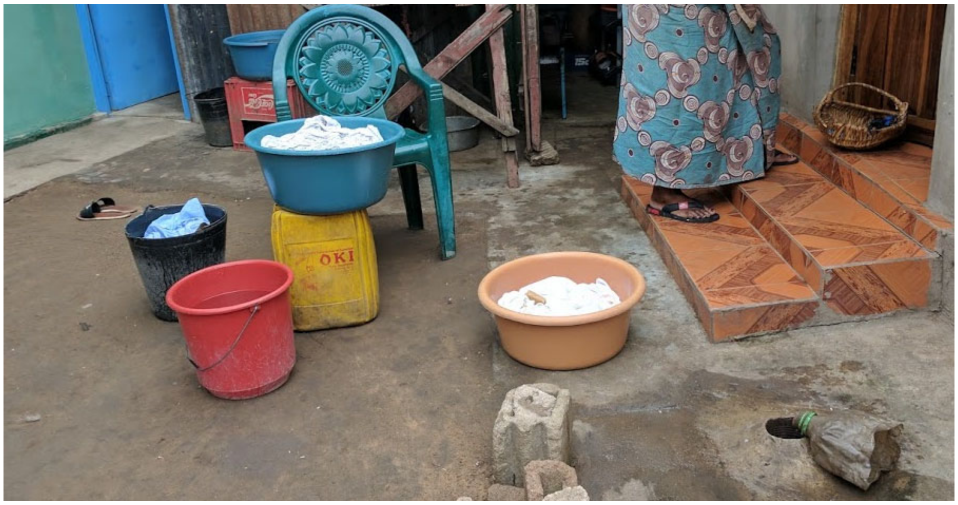

7. Example of the center of the compound yard

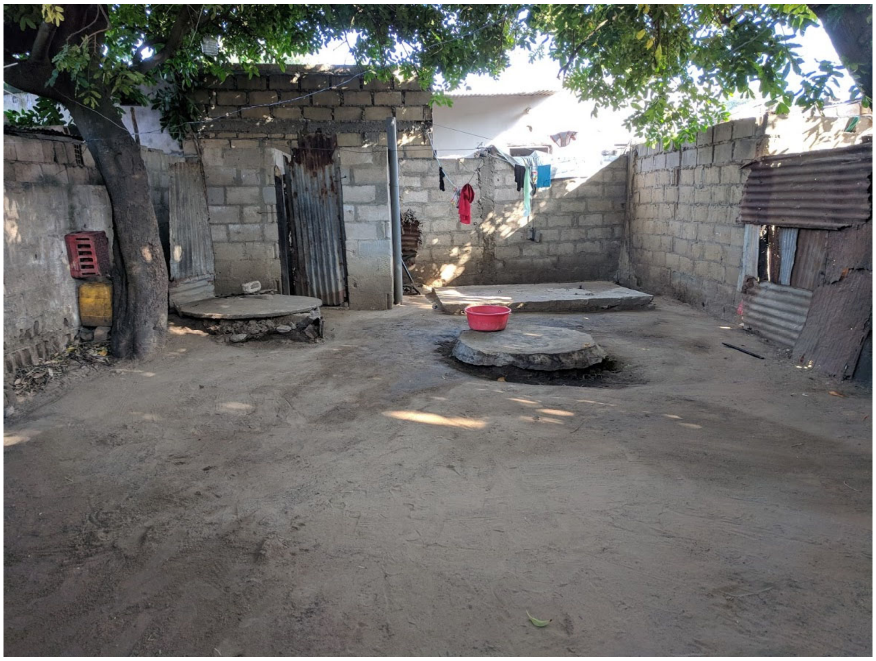

8. Aerial view of a neighborhood. Compounds are typically surrounded by walls made of block or corrugated metal. Compound entrances may be a gap in the walls, an empty doorway, or a lockable doorway.

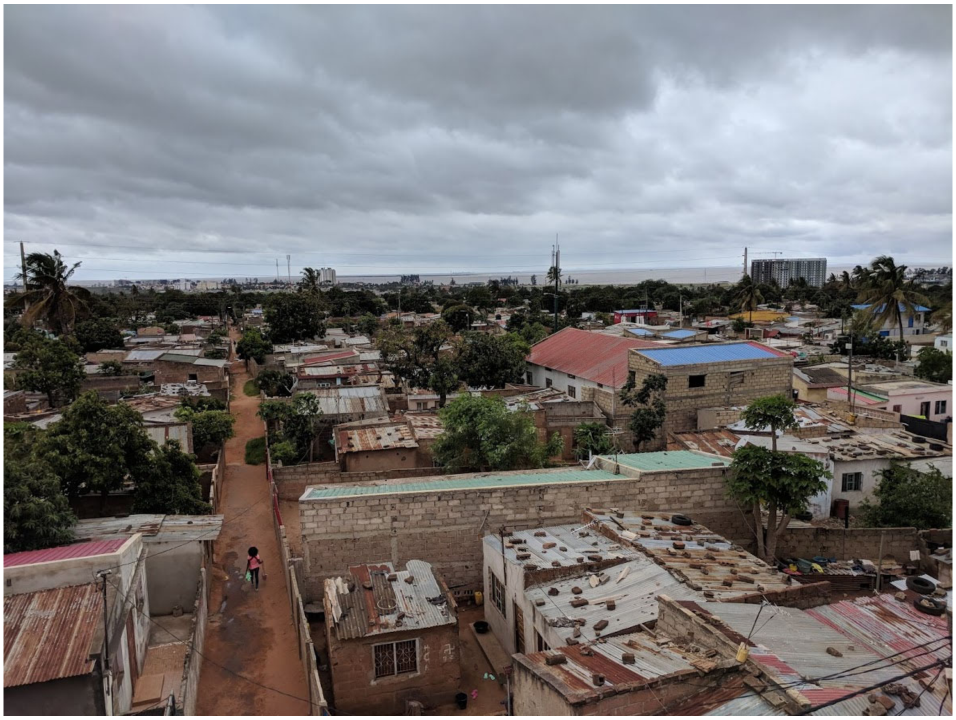

Supplement: S1 Fig — (PDF) [file pone.0224333.s001.pdf]
