# Supplementary material for: A localized sanitation status index as a proxy for fecal contamination in urban Maputo, Mozambique
Source: PLoS One. 2019 Oct 25;14(10):e0224333. doi: 10.1371/journal.pone.0224333 (PMC6814227; doi:10.1371/journal.pone.0224333)
Supplement: S1 Table — (PDF) [file pone.0224333.s002.pdf]

S1 Table. Sanitary Survey Inputs from Literature Review.

Variable Type	Milroy et. al	Mehta et. al	Campos et. al	Gunawardana et. al	Hawkins et. al	Jenkins et. al
**Socioeconomic variables**			X			
**Condition of habitation**	X		X			
**Extent of pavement**	X					
**Water access**	X	X	X	X	X	
**Fecal waste disposal chain variables**	X	X	X	X	X	
**Drainage & wastewater**	X		X	X	X	
**Solid waste collection**	X	X	X	X	X	
**Latrine sharing**			X	X	X	
**Latrine superstructure safety**					X	
**Hygiene**			X	x	X	
**Open defecation practices**						X
