# Supplementary material for: A localized sanitation status index as a proxy for fecal contamination in urban Maputo, Mozambique
Source: PLoS One. 2019 Oct 25;14(10):e0224333. doi: 10.1371/journal.pone.0224333 (PMC6814227; doi:10.1371/journal.pone.0224333)
Supplement: S1 Text — (PDF) [file pone.0224333.s003.pdf]

S1 Text. Additional LSSI Information.

LSSI Ordinal Weights

## Access to Infrastructure

*The type of toilet used by households and the number of households sharing it.*

**Access to infrastructure = 0.7 * Type of Toilet + 0.3 * Sharing toilet**

### Type of Toilet

| Classification | Score |
|---|---|
| Connection to a sewer system | 1 |
| Pour flush/squat pan connected to a septic tank or pit | 0.75 |
| Pit latrine with a concrete slab | 0.5 |
| Traditional pit latrine without a concrete slab | 0.25 |
| Open defecation, bucket, bag, etc. | 0 |

### On-site Sanitation Sharing

**Sharing toilet = 0.5 * Households + 0.5 * Persons**

Households

| | |
|---|---|
| 1 household (no sharing) | 1 |
| 2-3 households | 0.625 |
| 4-5 households | 0.125 |
| >5 households | 0 |

Persons

| | |
|---|---|
| 1-5 persons | 1 |
| 6-10 persons | 0.67 |
| 11-15 persons | 0.33 |
| >15 persons | 0 |

## Containment Safety

**Containment Safety = 0.25 * slab/floor stability + 0.125 * type of walls + 0.125 * type of roof + 0.25 * type of lining * groundwater level + 0.25 * containment effectiveness**

Slab/Floor Stability

| | |
|---|---|
| Overall in good condition and floor is made of concrete | 1 |

| | |
|---|---|
| Overall in good condition, but floor is made of sand | 0.75 |
| Cracked or broken, but no holes in the floor, and no risk of collapse | 0.5 |
| Cracked or broken, with holes in the floor, and no risk of collapse | 0.25 |
| Apparent risk of collapse | 0 |

Walls

| | |
|---|---|
| Masonry (blocks, concrete, etc.) | 1 |
| Corrugated iron sheets | 0.75 |
| Grass or reeds<br>OR Scrap Metal<br>OR Asbestos cement sheets | 0.5 |
| Plastic sheets, sacking, or cloth | 0.25 |
| None | 0 |

Roof

| | |
|---|---|
| Tiles<br>OR Concrete slab<br>OR Corrugated iron or asbestos cement sheets | 1 |
| Timber or scrap metal | 0.75 |
| Grass or reeds | 0.5 |
| Plastic sheets, sacks or cloth | 0.25 |
| None | 0 |

Lining

| | |
|---|---|
| Masonry | 1 |
| Tires | 0.75 |
| Timber / Wood | 0.5 |
| Metal or plastic barrels<br>OR Reeds | 0.25 |
| None | 0 |

Groundwater Level

| | |
|---|---|
| Low Water Table: >3 below ground level | 1 |
| Medium Water Table: 1.5-3 m below ground level | 0.5 |
| High Water Table: <1.5m below ground level | 0 |

Containment Effectiveness

| | |
|---|---|
| Liquid never overflows or leaks from the latrine or the ground near the latrine | 1 |
| Liquid rarely overflows or leaks from the latrine or the ground near the latrine | 0.667 |
| Liquid occasionally overflows or leaks from the latrine or the ground near the latrine | 0.333 |
| Liquid often overflows or leaks from the latrine or the ground near the latrine | 0 |

## Hygiene

**Hygiene = 0.4 * Hygienic condition + 0.3 * Soap + 0.3 * Cover**

Hygienic Condition

| | |
|---|---|
| Clean | 1 |
| Presence of dirty water | 0.75 |
| Presence of solid waste | 0.5 |
| Presence of urine | 0.25 |
| Presence of feces or used anal cleansing materials<br>OR Open defecation | 0 |

Soap

| | |
|---|---|
| Soap available, signs it has been used recently | 1 |
| Soap available, but no signs it has been used recently<br>No soap available, but signs it has been used recently | 0.5 |
| No soap available, no signs of recent use | 0 |

Cover

| | |
|---|---|
| Tight-fitting lid in place (latrine)<br>OR Water seal toilet (pour flush) | 1 |
| Loose fitting lid or cover in place (latrine) | 0.5 |
| No cover | 0 |

# Emptying and Transport

## Access to emptying services: future intentions for emptying on-site sanitation

Note: Where a latrine is covered over when full and rebuilt, the household is excluded from the calculation of this indicator and this component.

| | |
|---|---|
| Formal emptying: vacuum truck or trash pump<br>OR Sewer connection | 1 |
| Informal Emptying: buckets and other hand tools | 0 |

## Transport Safety

**If Local Transport = 0 (Transport = 0)**

**If Local Transport = 1 (Transport = 0.5 * Local Transport + 0.5 Neighborhood Transport)**

*Local Transport*

| | |
|---|---|
| Formal emptying and fecal sludge is taken to WWTP<br><br>OR Compound latrine will be covered and rebuilt | 1 |
| Compound empties sludge manually (ie. Buckets) and dumps into a hole in the yard, into a hole near the compound, or dumps in a nearby ditch or drain, | 0 |

Neighborhood Transport

| | |
|---|---|
| Formal emptying is most frequent in the nearby area (according to nearest *chefe de quarteirão)* | 1 |
| Local dumping of sludge occurs most frequently (according to nearest *chefe de quarteirão*). | 0 |

## Disposal

### Quality of Disposal Management

**If Disposal = Taken to WWTP (Final Disposal = 1)**

**If Disposal = Buried in-situ (Final Disposal = Disposal * Water Table)**

Disposal

| | |
|---|---|
| Fecal sludge taken to ETAR de Infulene (WWTP) | 1 |
| Buried in-situ | 0.667 |

Water Table

| | |
|---|---|
| Water table >3m deep | 1 |
| Water table 1.5-3m deep | 0.5 |
| Water table 1.5m or less | 0 |

## Complementary Services

### Water Access

| | |
|---|---|
| Water available in recipient at the latrine | 1 |
| Functioning tap within 1m of the latrine, but no recipient for when the water is not running | 0.5 |
| No water available | 0 |

## Solid Waste Management

**Solid waste = 0.8 * household solid waste + 0.2 * neighborhood solid waste**

### Household Solid Waste Management

| | |
|---|---|
| All solid waste is contained | 1 |
| There is some accumulation of solid waste inside the compound, but no large piles | 0.5 |
| There is significant accumulation of solid waste inside the compound | 0 |

### Neighborhood Solid Waste Management

| | |
|---|---|
| No accumulation of solid waste in the neighborhood | 1 |
| Accumulation of solid waste only around public waste skips | 0.5 |
| Significant accumulation of solid waste in public spaces | 0 |

## Storm and Grey Water Management

**Storm and Grey Water Management = 0.5 * Household Grey Water + 0.5 * Water Ponding**

### In-house Greywater Management

| | |
|---|---|
| Discharged to a drain<br>OR Used to water plants inside the compound | 1 |
| Poured into the latrine or septic tank | 0.5 |
| Dumped on the ground in the street or walkway<br>OR Dumped on the ground inside the compound | 0 |

### Local Accumulation of Storm Water

| | |
|---|---|
| After a rain storm standing water remains inside or near the compound for <1 day | 1 |
| After a rain storm standing water remains inside or near the compound for 1 day | 0.667 |
| After a rain storm standing water remains inside or near the compound for 2 days | 0.333 |
| After a rain storm standing water remains inside or near the compound for 3 or more days | 0 |
