# Supplementary material for: A localized sanitation status index as a proxy for fecal contamination in urban Maputo, Mozambique
Source: PLoS One. 2019 Oct 25;14(10):e0224333. doi: 10.1371/journal.pone.0224333 (PMC6814227; doi:10.1371/journal.pone.0224333)
Supplement: S2 Text — (PDF) [file pone.0224333.s004.pdf]

S2 Text. Survey questions in English and Portuguese

Question (English)	Question (Portuguese)	Response (Portuguese)	Response (English)
Quantos agregados familiares existem neste composto?	How many HHs live in this compound?	integer	
Quantas pessoas no total vivem neste composto (incluindo crianças)?	How many people in total live in this compound?	integer	
Quantas crianças menores de 5 anos vivem neste composto?	How many children less than 5 years of age live on this compound?	integer	
Alguém neste agregado possui algum desses seguintes itens? (escolha todos que se aplicarem)	Does anyone in your HH own the following items? BICYCLE	1. TRUE<br>0. FALSE	1. TRUE<br>0. FALSE
	Does anyone in your HH own the following items? MOTORBIKE	1. TRUE<br>0. FALSE	1. TRUE<br>0. FALSE
	Does anyone in your HH own the following items? CAR	1. TRUE<br>0. FALSE	1. TRUE<br>0. FALSE
	Does anyone in your HH own the following items? IRON	1. TRUE<br>0. FALSE	1. TRUE<br>0. FALSE
	Does anyone in your HH own the following items? FREEZER	1. TRUE<br>0. FALSE	1. TRUE<br>0. FALSE
	Does anyone in your HH own the following items? CLOCK	1. TRUE<br>0. FALSE	1. TRUE<br>0. FALSE
	Does anyone in your HH own the following items? RADIO	1. TRUE<br>0. FALSE	1. TRUE<br>0. FALSE
	Does anyone in your HH own the following items? STEREO	1. TRUE<br>0. FALSE	1. TRUE<br>0. FALSE
	Does anyone in your HH own the following items? CASSETTE PLAYER	1. TRUE<br>0. FALSE	1. TRUE<br>0. FALSE
	Does anyone in your HH own the following items? SOFA	1. TRUE<br>0. FALSE	1. TRUE<br>0. FALSE
	Does anyone in your HH own the following items? NONE OF THE ABOVE	1. TRUE<br>0. FALSE	1. TRUE<br>0. FALSE

Onde o seu agregado normalmente colecta água?	Where does your HH normally collect water from?	1. Torneira em casa/ torneira privada<br>2. Torneira no composto<br>3. Torneira do vizinho<br>4. Torneira ou fontanário público<br>5. Poço protegido<br>6. Poço desprotegido<br>7. Água da chuva<br>8. Furo	1. Tap in the home<br>2. Tap outside the home<br>3. Neighbours tap<br>4. Public tap<br>5. Protected spring<br>6. Unprotected spring<br>7. Rainwater<br>8. Borehole
Quantas horas por dia a água esta disponível na fonte?	How many hours a day is water available?	1. <1 hora<br>2. 1-3 horas<br>3. 4-6 horas<br>4. 7-8 horas<br>5. > 8 horas	1. <1 hr<br>2. 1-3 hrs<br>3. 4-6 hrs<br>4. 7-8 hrs<br>5. > 8 hrs
Quantas latrinas construídas pela WSUP existem no composto?	How many WSUP latrines are there in this compound?	integer	
Que tipo de latrina da WSUP existe no composto?	Which type of WSUP latrine is there on this compound?	Blocos sanitarios<br>Latrina partilhada	
Quantas latrinas não construídas pela WSUP existem no composto?	How many non-WSUP latrines are there in this compound	integer	
Para cada tipo de latrinas / WC, quantos existem no composto : Latrina da WSUP - Number	How many WSUP latrines are there on this compound?	integer	
Para cada tipo de latrinas / WC, quantos existem no composto : Despejo para sistema de esgoto - Number	How many latrines connected to the sewer are there on this compound?	integer	
Para cada tipo de latrinas / WC, quantos existem no composto : Despejo para fossa séptica - Number	How many latrines connected to a septic tank are there on this compound?	integer	
Para cada tipo de latrinas / WC, quantos existem no composto : Despejo manual para fora ou fossa	How many latrines: flush / pour flush to onsite / above ground pit	integer	

superrficial - Number			
Para cada tipo de latrinas / WC, quantos existem no composto : Latrina com laje de concreto - Number	How many latrines: pit with concrete slab	integer	
Para cada tipo de latrinas / WC, quantos existem no composto : Latrina sem laje de concreto - Number	How many latrines: pit without concrete slab	integer	
Para cada tipo de latrinas / WC, quantos existem no composto : Balde - Number	How many latrines/sites: bucket	integer	
Para cada tipo de latrinas / WC, quantos existem no composto : Plástico - Number	How many latrines/sites: plastic bag	integer	
Para cada tipo de latrinas / WC, quantos existem no composto : Fecalismo a ceu aberto - Number	How many sites: open defecation	integer	
Tire uma foto da latrina	Take a photo of the latrine	photo	
Observe: De que material é feito o piso das latrinas/sanitário?	Observe: What material is the latrine floor made of?	0. Piso de terra<br>1. Bloco de concreto<br>2. Laje de concreto<br>3. Madeira<br>4. Tijoleira<br>20. Other	0. Dirt floor<br>1. Concrete block<br>2. Concrete slab<br>3. Wood<br>4. Scrap metal<br>20. Other:

Observe: De que material são construídas as paredes das latrinas/ sanitário?	*Observe:* What material are the latrine walls built from?	1. Alvenaria<br>2. Chapa de zinco<br>3. Tijolos de lama<br>4. Madeira<br>5. Ferro velho<br>6. Folhas de cimento<br>7. Capim ou caniço<br>8. Plástico, sacos ou pano<br>9. Latrinas sem paredes<br>20. Other	1. Masonry<br>2. Corrugated iron sheets<br>3. Mud bricks<br>4. Timber<br>5. Scrap metal<br>6. Cement sheets<br>7. Grass or reeds<br>8. Plastic sheets, sacking or cloth<br>9. No latrine walls<br>20. Other:
Observe: De que material é construído a cobertura das latrinas?	*Observe:* What material is the latrine roof built from?	1. telhas<br>2. laje de concreto<br>3. chapos de zinco<br>4. madeira<br>5. ferro velho<br>6. capim ou caniço<br>7. plástico, sacos ou pano<br>8. nenhum<br>20. Other	1. Tiles<br>2. concrete slab<br>3. corrugated iron sheets<br>4. timber<br>5. scrap metal<br>6. Grass or reeds<br>7. Plastic sheets, sacking or cloth<br>8. None<br>20. Other:
Observe/pergunte: Qual a condição da laje / piso?	*Observe/ask: What* is the condition of the slab / floor?	1. Em geral, em boas condições<br>2. Rachados ou quebrado, mas sem furos no chão ou risco aparente de colapso<br>3. Rachados ou quebrados, buracos no chão, mas sem risco de colapso<br>4. Laje ou piso parece em risco de colapso<br>5. Em geral apresenta boas condições, mas sem laje de concreto, o piso é de areia	1. Overall in good condition<br>2. Cracked or broken, but with no holes in the floor or apparent risk of collapse<br>3. Cracked or broken, holes in the floor, but with no risk of collapse<br>4. Slab or floor appears at risk of collapse<br>5. In general appears in good condition but there is no concrete slab
Observe: A água está disponível perto da latrina / sanita?	*Observe:* Is water available near the latrine/toilet?	1. A água está disponível num recipiente<br>2. A água está disponível na torneira ao lado da latrina	1. Water is available in a container<br>2. Water is available at tap next to the latrine<br>3. Water is not available

		3. A água não está disponível	
Observe: Existe acumulação de lixo dentro do composto?	Observe: Is there garbage accumulation inside this compound?	2. Sim, há montes de lixo<br>1. Sim. Não há montes de lixo, mas há algum lixo acumulado em volta do composto<br>0. Não, todo o lixo está contido	2. Yes, there are piles of garbage<br>1. Yes. There are no piles of garbage, but there is some accumulation of garbage around the compound<br>0. No, all garbage is contained
Observe: Actualmente há água estagnada neste composto?	*Observe:* Is there currently standing water at this compound?	0. Nao<br>1. Sim	
Observe: Qual é a condição higiénica desta latrina?	Observe: What is the hygienic condition of this latrine? Presence of dirty water	1. TRUE<br>0. FALSE	
	Observe: What is the hygienic condition of this latrine? Presence of solid waste	1. TRUE<br>0. FALSE	
	Observe: What is the hygienic condition of this latrine? Presence of urine	1. TRUE<br>0. FALSE	
	Observe: What is the hygienic condition of this latrine? Presence of anal cleaning materials used	1. TRUE<br>0. FALSE	
	Observe: What is the hygienic condition of this latrine? Presence of feces	1. TRUE<br>0. FALSE	
	Observe: What is the hygienic condition of this latrine? Nothing observed: the latrine looks clean and well maintained	1. TRUE<br>0. FALSE	

Observe: Como é que o buraco está coberto?	*Observe:* How is the drop hole covered?	1. Tampa ajustada no lugar (latrina)<br>2. Tampa flexível no lugar (latrina)<br>3. Sem cobertura<br>4. Selagem de água (despejo)<br>5. Selagem de água quebrada ou não funcional (despejo)<br>20. Other	1. Tight fitting lid in place (latrine)<br>2. Loose fitting lid or cover in place (latrine)<br>3. No cover<br>4. Water-seal (pour-flush)<br>5. Broken or non-functional water-seal (pour flush)<br>20. Other:
PERGUNTE: de que material foi feito o revestimento da fossa ou fossa septica?	Ask: What is the septic tank made from?	1. Alvenaria<br>2. Conectado ao sistema de esgotos<br>3. Pneus<br>4. Madeira<br>5. Barris de metal ou plástico<br>6. Caniço<br>7. Nenhum<br>20. Other	1. Masonry<br>2. Connected to sewerage system<br>3. Tires<br>4. Timber<br>5. Metal or plastic barrels<br>6. Reeds<br>7. None<br>20. Other
Pergunte: Com que frequência líquidos transbordam ou escorrem desta latrina ou para o chão?	Ask: How often does liquid leak from this latrine or seep up through the ground near the latrine?	1. Nunca<br>2. Muito raro<br>3. Ocasionalmente<br>4. Frequentemente	1. Never<br>2. Very rarely<br>3. Occasionally<br>4. Often
Por favor tire uma foto do local onde os membros do agregado normalmente lavam as mãos	Take a photo of the place where HH members usually wash their hands	photo	
Este é o mesmo local onde lava as mãos depois de usar a latrina?	Is this the same place where you wash your hands after using the toilet?	0. Nao<br>1. Sim	
OBSERVE Instalação fixa observada (Torneira de lavatório)	Where is the HWF located?	1. Na habitação<br>2. No pátio / quintal<br>3. Ao lado da latrina / sanitário<br>4. Objecto móvel observado (balde / jarro / chaleira)<br>5. Nenhuma instalação de lavagem das mãos observada	1. In dwelling<br>2. In yard /plot<br>3. Next to latrine/toilet<br>4. Mobile object observed (Bucket / Jug / Kettle)<br>5. No hand washing facility observed

A água está disponível nas instalações de lavagem das mãos?	Is there water avialble at the HWF to wash hands?	0. Nao<br>1. Sim	
Há presença de sabão / detergente / água com sabão no local para lavagem das mãos? (1)	Is there soap/ detergent or soapy water present at the place for HW?	0. Nao<br>1. Sim	
Existem sinais de que o sabão tenha sido usado recentemente nesta instalação de lavagem das mãos (por exemplo, resíduo de sabão) (1)	Are there any signs that soap has been used recently at this handwashing facility (e.g. soap residue)	0. Nao<br>1. Sim	
Por favor tire uma foto do local onde os membros do agregado lavam as mãos após usar a latrina/sanitário	Take a photo of the place where HH members wash their hands after using the toilet	photo	
Tem água disponível nessa instalação de lavagem das mãos?	is there water available to wash hands?	0. Nao<br>1. Sim	
Há presença de sabão / detergente / água com sabão no local para lavagem das mãos? (2)	Is there soap/ detergent or soapy water present at the place for HW?	0. Nao<br>1. Sim	
Existem sinais de que o sabão tenha sido usado recentemente nesta instalação de lavagem das mãos (por exemplo, resíduo de sabão) (2)	Are there any signs that soap has been used recently at this handwashing facility (e.g. soap residue)	0. Nao<br>1. Sim	
Que tipo de latrina as pessoas da sua casa mais usam?	What type of latrine do people in your HH use most often?	1. Latrina da WSUP<br>2. Despejo para sistema de esgoto<br>3. Despejo para fossa séptica<br>4. Despejo manual para fora ou fossa superrficial<br>5. Latrina com laje de concreto<br>6. Latrina sem laje de concreto<br>7. Balde<br>8. Plástico<br>9. Fecalismo a ceu aberto	1. WSUP latrine<br>2. flush / pour flush toilet to sewer<br>3. flush / pour flush to underground tank<br>4. flush / pour flush to onsite / above ground pit<br>5. pit with concrete slab<br>6. pit without concrete slab<br>7. bucket<br>8. bag<br>9. open defecation

Compartilha a latrina que usa frequentemente com outras famílias?	Do you share the latrine/toilet you use most often with other families?	0. Nao<br>1. Sim	
A latrina privada possui as seguintes condições? (marque todos os que se aplicam)	Private latrine has the following attributes (tick all that apply) Cleanable concrete, tile, or masonry slab	1. TRUE<br>0. FALSE	
	Private latrine has the following attributes (tick all that apply) Masonry tile, concrete, or pedestal	1. TRUE<br>0. FALSE	
	Private latrine has the following attributes (tick all that apply) Formal latrine superstructure	1. TRUE<br>0. FALSE	
	Private latrine has the following attributes (tick all that apply) Covered ventilation tube	1. TRUE<br>0. FALSE	
Compartilha todas as latrinas / buracos com todos os outros membros do composto ou determinadas latrinas são designadas para uso de determinadas famílias / gêneros / outros grupos?	Do you share all latrine stalls/drop holes with all other compound members or are certain stalls/drop holes designated for use by certain households/genders/other groups?	1. Todos os membros do composto compartilham a mesma latrina / sanitário<br>2. Minha casa usa um banheiro / latrina específica<br>3. Latrinas / sanitários são separados por gênero<br>4. Latrinas / sanitários são separados de acordo com as famílias	1. All compound members share the same latrine/toilet<br>2. My household uses a specific toilet/latrine<br>3. Latrine/toilets are separated by gender 4. Latrine/toilets are separated by families
Quantas famílias compartilham a latrina / sanitário que usa frequentemente?	How many families share the latrine/ toilet you use most often?	0. Apenas seu agregado<br>1. 2- 3 agregados<br>2. 4 – 5 agregados<br>3. 5 – 10 agregados<br>4. Mais 10 agregados	0. Just your household<br>1. 2- 3 households<br>2. 4 – 5 households<br>3. 5 – 10 households<br>4. More than 10 households

Quantas pessoas, incluindo a si e as crianças, compartilham a latrina / sanitário que usa frequentemente?	How many people including children share the latrine /toielt you use most often?	0. 1- 5 pessoas<br>1. 6-10 pessoas<br>2. 11 – 15 pessoas<br>3. 16 – 20 pessoas<br>4. Mais de 20 pessoas	0. 1- 5 people<br>1. 6-10 people<br>2. 11 – 15 people<br>3. 16 – 20 people<br>4. More than 20 people
Qualquer pessoa (publico em geral) pode usar a latrina / sanitário que usa frequentemente?	Can anyone (general public) use the toilet you use most often?	0. Nao<br>1. Sim	
Estava a viver neste composto quando a latrina / sanitário que usa frequentemente foi construído?	Were you living on this compound when the toilet/ latrine you use most often was constructed?	0. Nao<br>1. Sim	
O que aconteceu com a latrina que usava antes?	What happened to the latrine you used before?	1. Abandonou<br>2. Coberto com terra<br>3. Coberto com lixo<br>4. Coberto com outros<br>5. Vazio<br>20. Other	1. Abandoned<br>2. Covered with earth<br>3. Covered with garbage<br>4. Covered with other<br>5. Emptied<br>20. Other
Em que ano os residentes deste composto começaram a usar a latrina / sanitário que usa frequentemente?	In what year did the residents of this compound begin to use the latrine/ toilet you use most often?	integer (MT)	
Em que época do ano?	In what part of the year?	1. Inícios<br>2. Meados<br>3. Finais	
Quantas vezes foi construída uma nova fossa neste composto nos últimos 3 anos?	How many times has a new septic tank been contructed in this compound in the last 3 years	integer (MT)	
Quantas vezes foi esvaziada uma latrina / fossa séptica neste composto nos últimos 3 anos?	How many times has a latrine/septic tank on this compound been emptied in the last three years?	integer (MT)	

Quando foi a última vez que a latrina / fossa séptica que usa frequentemente foi esvaziada?	When was the last time the latrine/septic tank that you use most often was emptied?	1. Nunca<br>2. Na última semana<br>3. No último mês<br>4. "Nos últimos seis meses "<br>5. "No último ano "<br>6. Nos últimos dois anos<br>7. Nos últimos 5 anos<br>8. Há mais de cinco anos<br>9. Pit / fossa séptica foi substituída, não foi esvaziada<br>20. Other	1. Never<br>2. With the last week<br>3. Within the last month<br>4. Within the last six months<br>5. Within the last year<br>6. Within the last two years<br>7. Within the last 5 years<br>8. More than five years ago<br>9. Pit/ tank was replaced not emptied<br>20. Others: ___________
Quais destes serviços de esvaziamento estão disponíveis no seu bairro?	Which of these services exist in your barrio? Serviço de esvaziamento manual (ex. sacos)	"1. TRUE<br>0. FALSE"	
	Which of these services exist in your barrio? Bomba de esvaziamento manual	1. TRUE<br>0. FALSE	
	Which of these services exist in your barrio? Bomba de esvaziamento mecânica	1. TRUE<br>0. FALSE	
	Which of these services exist in your barrio? Esvaziamento mecânico a vácuo	1. TRUE<br>0. FALSE	
Na ultima vez, quem esvaziou a fossa da latrina que usa frequentemente?	last time, who emptied the septic tank you use most frequently?	1. Latrina / fossa séptica foi esvaziada por membro AF<br>2. Latrina / fossa séptica foi esvaziada por Esvaziador informal<br>3. Latrina / fossa séptica foi esvaziada por um esvaziador formal<br>20. Other	1. HH member<br>2. Informal emptier<br>3. Formal emptier<br>20. Other

A última vez que a latrina ou fossa séptica que usa frequentemente foi esvaziada, qual foi o equipamento usado para esvaziar?	Last time the latrine/septic tank you use most frequently was emptied what equipment was used to empty it?	1. À mão, usando sacos, baldes ou ferramentas manuais semelhantes<br>2. À mão, usando uma bomba manual (como um gulper)<br>3. Mecanicamente, usando uma pequena bomba mecânica<br>4. Mecanicamente, usando um caminhão de vácuo<br>5. Incerto	1. By hand, using sacks, buckets or similar hand tools<br>2. By hand, using a hand pump (such as a gulper)<br>3. Mechanically, using a small mechanical pump (such as a trash pump)<br>4. Mechanically, using a vacuum truck<br>5. Unsure
Por que não escolheu usar um serviço de esvaziamento formal a última vez que esvaziou a sua fossa?	Why did you not choose a formal emptying service the last time you emptied the tank	0. Custo<br>1. O serviço não chega a minha casa<br>20. Other	0. Cost<br>1. Service not avilable near my house<br>20. Other
A última vez que a latrina ou fossa séptica que usa frequentemente foi esvaziada, qual era o nome da empresa que esvaziou?	Last time the latrine/septic tank you use most frequently was emptied which service provider did you use?	1. ACADEC<br>2. Mbonga Mbilo<br>3. Limpezas Maguanine<br>4. Oliveiras<br>5. Sisema<br>6. Paulinho<br>7. Município<br>20. Other	
A última vez que a latrina ou fossa séptica que usa frequentemente foi esvaziada, onde foram descartados os resíduos fecais?	Last time the pit or septic tank you use most often was emptied, where was the fecal waste disposed?	1. Enterrado dentro do composto<br>2. Enterrado nas proximidades, mas não dentro do composto<br>3. Deitado no chão fora do composto<br>4. Levado para ETAR de Infulene<br>5. Deitado fora do composto (destino desconhecido)<br>6. Deitado fora do composto (please specify)<br>7. Parte incerta<br>20. Other	1. Buried inside the compound<br>2. Buried nearby, but not inside the compound<br>3. Dumped on the ground nearby the compound<br>4. Taken to “O ETAR de Infulene<br>5. Taken outside the compound (destination unknown)<br>6. Taken outside the compound (specify destination) ________<br>7. Uncertain<br>20. Others: ___________

A última vez que a latrina ou fossa séptica que usa frequentemente foi esvaziada, onde foram descartados os resíduos fecais? (Deitado fora do composto (please specify)) - specify	Last time the pit or septic tank you use most often was emptied, where was the fecal waste disposed? (if outside of compound specify desitination)	text	
A última vez que a latrina ou fossa séptica que usa frequentemente foi esvaziada, onde foram descartados os resíduos fecais? (Other (please specify)) - specify	Last time the pit or septic tank you use most often was emptied, where was the fecal waste disposed? (other)	text	
A última vez que a latrina ou fossa séptica que usa frequentemente foi esvaziada, onde foram descartados os resíduos fecais? (Not Applicable)	Last time the pit or septic tank you use most often was emptied, where was the fecal waste disposed? (n/a)	1. TRUE<br>0. FALSE	
A última vez que a latrina ou fossa séptica que usa frequentemente foi esvaziada, o que foi usado para limpar a área e o equipamento depois?	Last time the pit or septic tank you use the most often was emptied, what was used to clean the area and equipment afterwards? Nothing	1. TRUE<br>0. FALSE	
	Last time the pit or septic tank you use the most often was emptied, what was used to clean the area and equipment afterwards? Water	1. TRUE<br>0. FALSE	
	Last time the pit or septic tank you use the most often was emptied, what was used to clean the area and equipment afterwards? Bleach	1. TRUE<br>0. FALSE	
	Last time the pit or septic tank you use the most often was emptied, what was used to clean the area and equipment afterwards? Soap (Omo)	1. TRUE<br>0. FALSE	

	Last time the pit or septic tank you use the most often was emptied, what was used to clean the area and equipment afterwards? Disinfectant	1. TRUE<br>0. FALSE	
	Last time the pit or septic tank you use the most often was emptied, what was used to clean the area and equipment afterwards? Petrol	1. TRUE<br>0. FALSE	
	Last time the pit or septic tank you use the most often was emptied, what was used to clean the area and equipment afterwards? Salt	1. TRUE<br>0. FALSE	
	Last time the pit or septic tank you use the most often was emptied, what was used to clean the area and equipment afterwards? Other	1. TRUE<br>0. FALSE	
A próxima vez que a fossa ficar cheia, que acções especificas espera que sejam realizadas?	The next time you need to empty a pit or septic tank on this compound, what specific action do you expect to be performed?	1. Tapar e abrir uma outra dentro do composto<br>2. Tapar e usar a latrina de um outro composto<br>3. Latrina / fossa séptica será esvaziada por membro AF<br>4. Latrina / fossa séptica será esvaziada por Esvaziador informal<br>5. Latrina / fossa séptica será esvaziada por um esvaziador formal<br>6. Latrina / fossa séptica será substituída, não foi esvaziada<br>20. Other	1. close and open another pit in the compound<br>2. Close and use neighbour's latrine<br>3. Latine/ Septic tank will be emptied by a HH member<br>4. Latrine/ septic tank will be emptied by informal emptier<br>5. Latrine/ Septic tank will be emptied by formal emptier<br>6. Latrine/ septic tank will be replaced not emptied<br>20. Other

Na próxima vez que uma latrina/ fossa séptica deste composto necessite de esvaziamento, onde espera que os resíduos fecais sejam descartados?	Next time a pit or septic tank on this compound is emptied, where do you expect the fecal waste to be disposed?	1. Enterrado dentro do composto<br>2. Enterrado nas proximidades, mas não dentro do composto<br>3. Deitado no chão fora do composto<br>4. Levado para ETAR de Infulene<br>5. Deitado fora do composto (destino desconhecido)<br>6. Deitado fora do composto (please specify)<br>7. Parte incerta<br>8. Nunca esvaziar<br>20. Other	
Na próxima vez que substituir uma latrina desse composto, o que fará com a latrina antiga?	Next time you replace a latrine on this compound, what will you do with the old latrine?	1. Abandonar<br>2. Cubrir com sujeira/lixo<br>3. Cubrir com _______ (especifique)<br>20. Other	
Na próxima vez que as pessoas desse composto decidirem substituir uma latrina, como determinarão que a latrina / sanitário precisa ser substituído?	Next time people on this compound decide to replace a latrine, how will they determine the latrine/toilet needs to be replaced?	1. Cheiro<br>2. Inspeção visual do nível das lamas fecais<br>3. Passado um certo prazo<br>4. Latrina / WC estiver transbordando<br>5. A estrutura de latrina / sanitário estiver danificado<br>20. Other	
Na próxima vez que as pessoas desse composto decidirem substituir uma latrina, como determinarão que a latrina / sanitário precisa ser substituído? (Other (please specify)) - specify	Next time people on this compound decide to replace a latrine, how will they determine the latrine/toilet needs to be replaced? (other)	text	

Na próxima vez que uma latrina neste composto for substituída, qual será o principal motivo para a escolha de substituir em vez de esvaziar?	Next time a latrine on this compound is replaced, what will be the main reason for choosing to replace instead of emptying?	1. Custo<br>2. Acesso<br>3. Conveniência<br>4. Simplicidade<br>5. Saúde<br>6. Segurança<br>20. Other	
O que faz com mais frequência com as águas negras?	What do you normally do with grey water?	1. Despeja no chão dentro do composto<br>2. Despeja no chão na rua<br>3. Usa para regar plantas ou árvores dentro do composto<br>4. Despeja num dreno<br>5. Despeja na latrina ou no sanitário<br>20. Other	
O que faz com mais frequência com as águas negras? (Other (please specify)) - specify	What do you normally do with grey water? (other)	text	
Em geral, por quantos dias após chover há agua estagnada no composto?	In general, how many days per year is there standing water inside the compound?	integer (MT)	
No último ano, alguma latrina ou fossa séptica deste composto transbordou sua estrutura de contenção?	In the last year, has any pit or septic tank on this compound overflowed its containment structure?	0. Nao<br>1. Sim	
