# Supplementary material for: A localized sanitation status index as a proxy for fecal contamination in urban Maputo, Mozambique
Source: PLoS One. 2019 Oct 25;14(10):e0224333. doi: 10.1371/journal.pone.0224333 (PMC6814227; doi:10.1371/journal.pone.0224333)

S3 Text. Descriptive definitions.

Visibly moist soil: Wet soil is darker in color than dry soil.

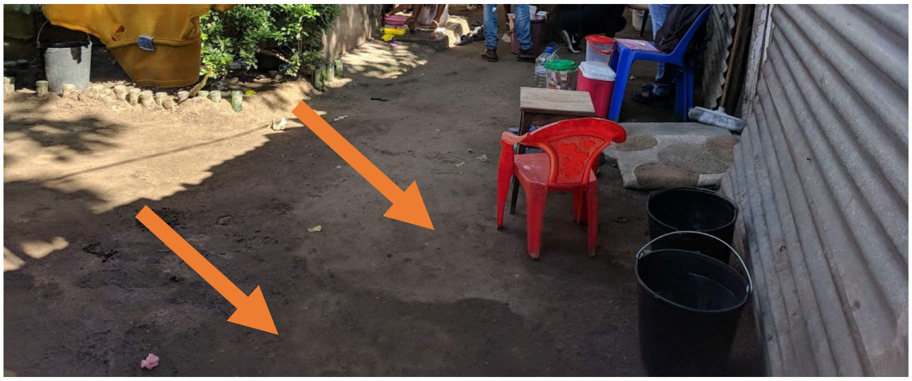

Full shade: An example of "full shade" underneath a covering. Fully shaded sample locations received no direct sunlight from 9:00am to 3:00pm.

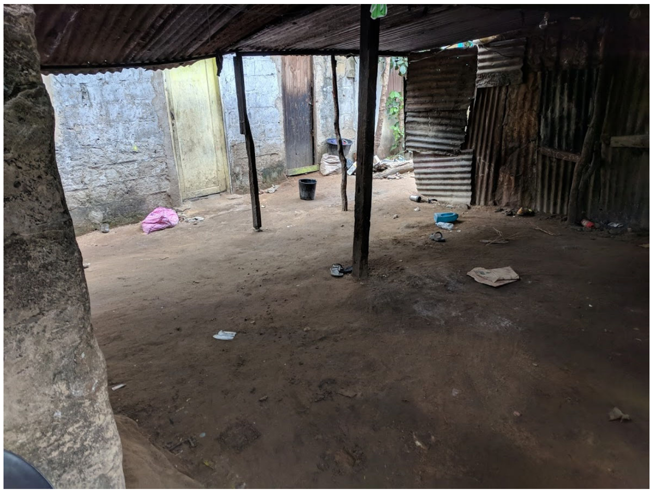

Full sun: An example of "full sun". Samples in full sun were out in the open, not near any trees or coverings that could provide shade. Completely sunny sample locations received direct sunlight from 9:00am to 3:00pm.

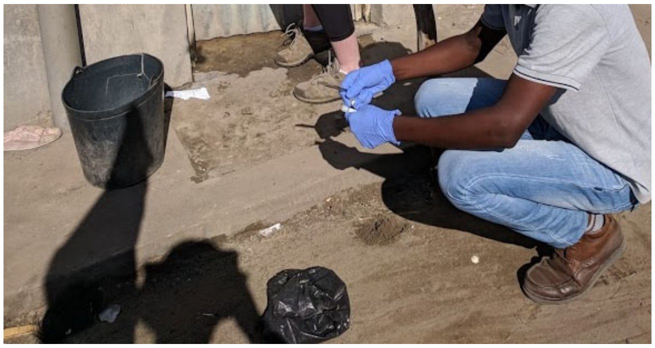

<u>Partially shaded/sunny</u>: An example of an area that is partially shaded. As the sun moves from 9:00am to 3:00pm this area received both direct sunlight and complete shade.

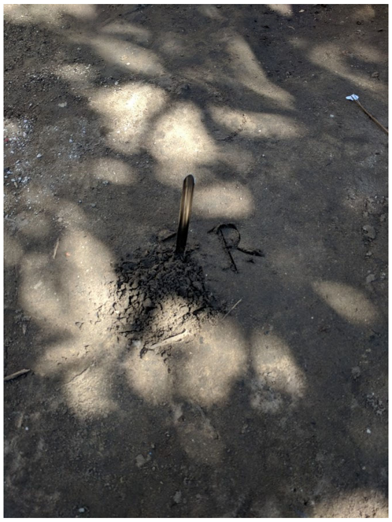

Supplement: S3 Text — (PDF) [file pone.0224333.s005.pdf]
