# Supplementary figures and images for: A localized sanitation status index as a proxy for fecal contamination in urban Maputo, Mozambique
Source: PLoS One. 2019 Oct 25;14(10):e0224333. doi: 10.1371/journal.pone.0224333 (PMC6814227; doi:10.1371/journal.pone.0224333)

S2 Fig. Map of project area.

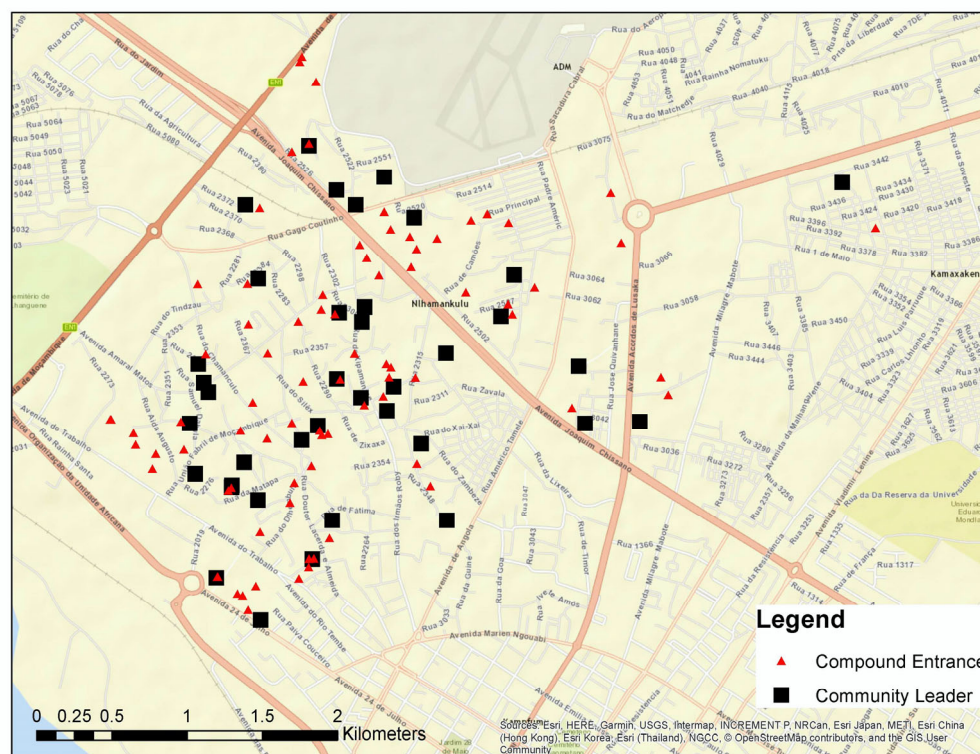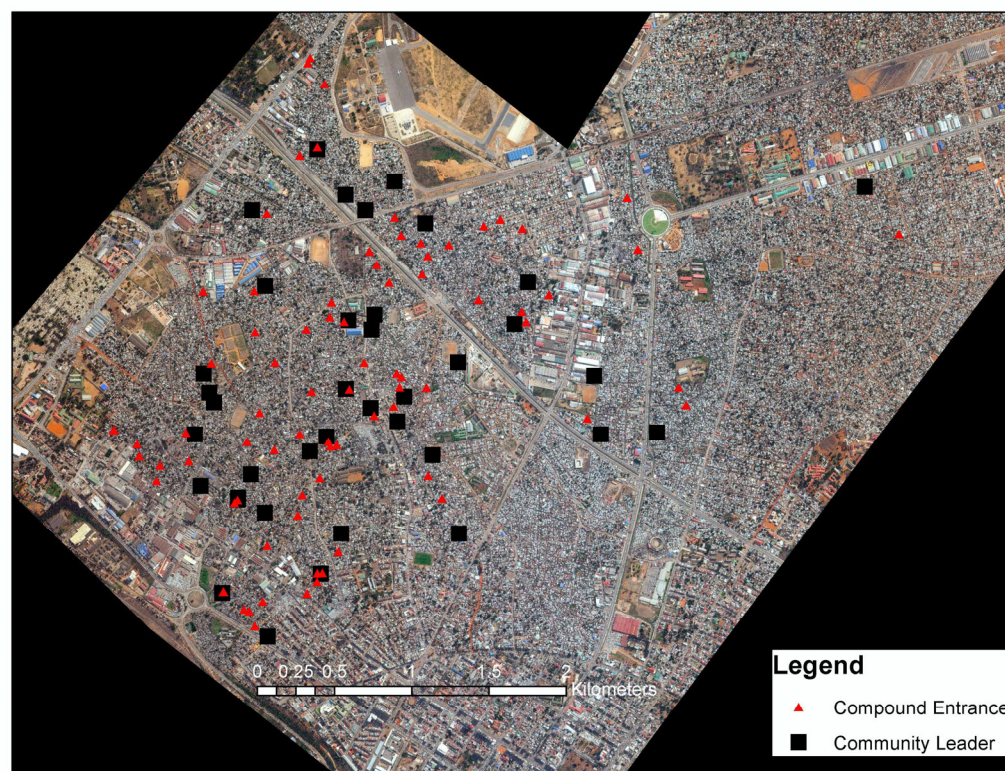

Supplement: S2 Fig — (PDF) [file pone.0224333.s006.pdf]

S4 Fig. Directed acyclic graph for model selection.

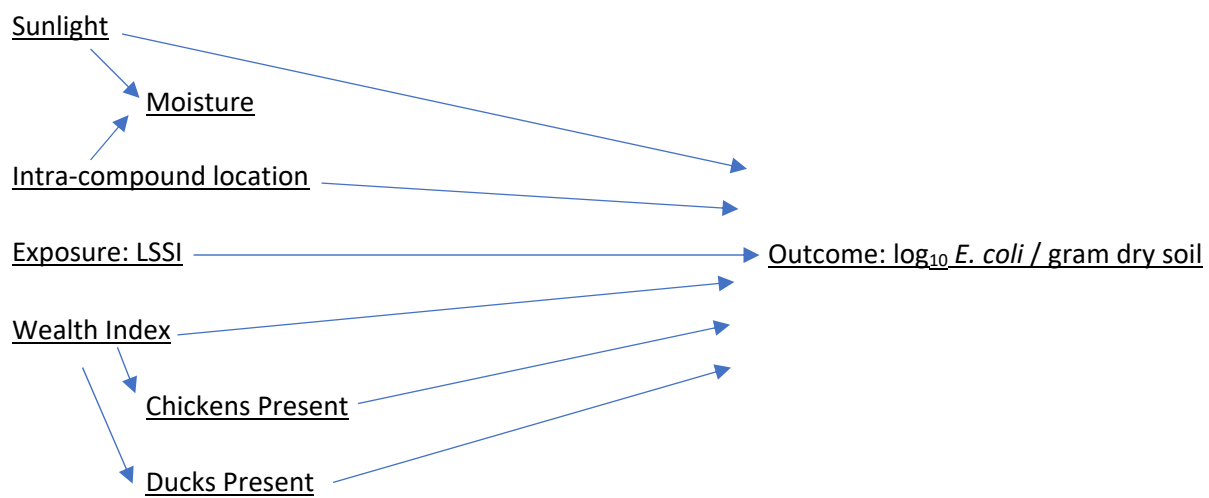

Supplement: S4 Fig — (PDF) [file pone.0224333.s009.pdf]

S5 Fig. LSSI vs *E. coli* counts scatterplots.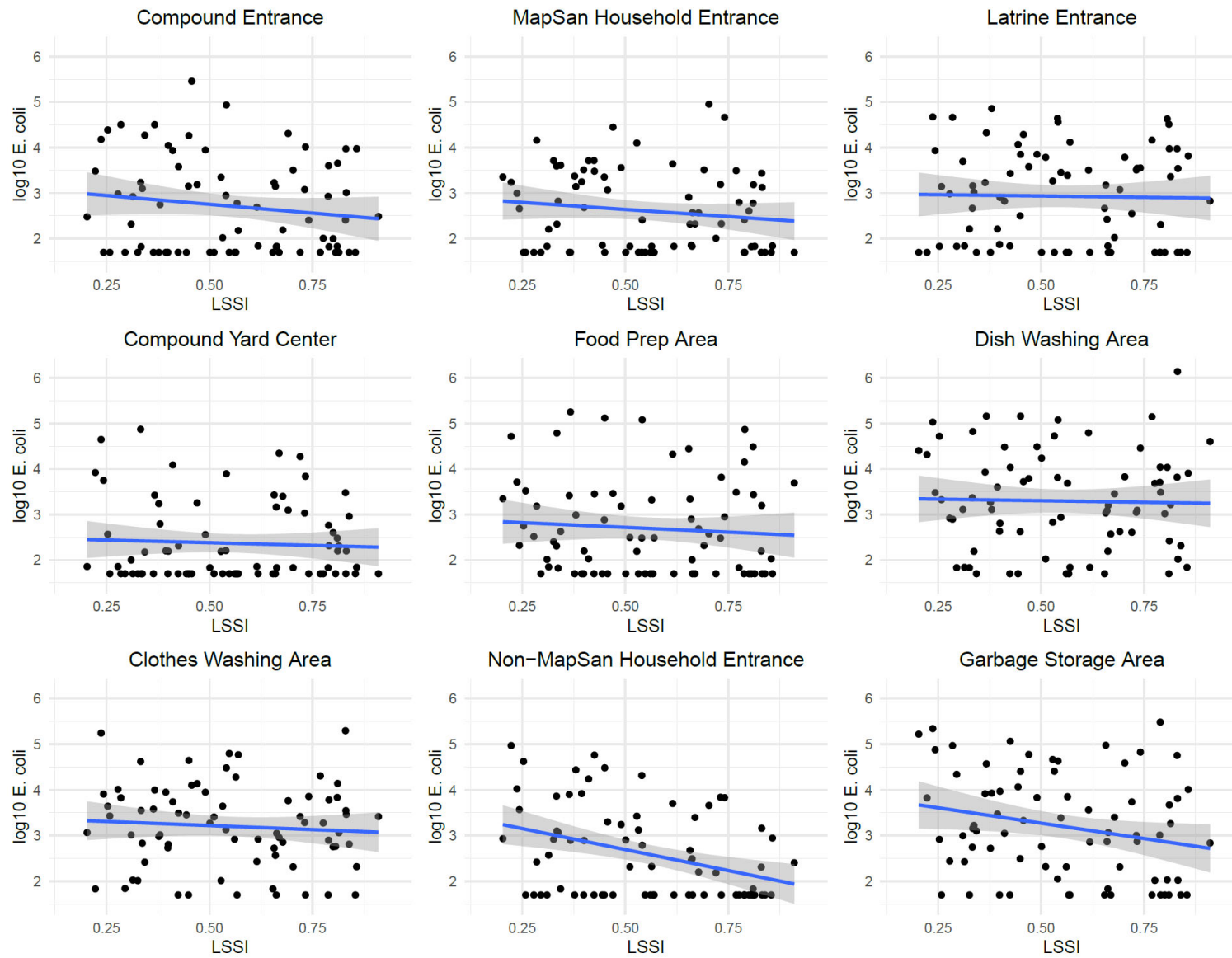

Supplement: S5 Fig — (PDF) [file pone.0224333.s014.pdf]
