# Supplementary material for: A localized sanitation status index as a proxy for fecal contamination in urban Maputo, Mozambique
Source: PLoS One. 2019 Oct 25;14(10):e0224333. doi: 10.1371/journal.pone.0224333 (PMC6814227; doi:10.1371/journal.pone.0224333)
Supplement: S4 Text — (PDF) [file pone.0224333.s007.pdf]

S4 Text. Validation of Ojal *E. coli* Test Kit.

We swabbed an additional 100 cm^2 surface on 33 of the 80 plastic chairs we tested as part of the study to validate the Ojal test kit. We followed the same swab procedure, except both wet and dry swabs were stored in a 15mL centrifuge tube containing 3ml of sterile ringer's solution (Sigma Aldrich, St. Louis, Missouri) and shaken manually for 2 minutes. Samples were placed on ice and within 6 hours of collection we plated a 1 mL aliquot of the ringer's solution onto a Compact Dry EC plate and incubated the plates for 24 hours at 37°C as per manufacturer's instructions.

Using the Compact Dry EC plates, we found 15% of surfaces tested positive for the presence of *E. coli* and 5.2% surfaces tested positive for *E. coli* at concentrations ≥10 *E. coli* per 100cm^2. The 15% detection of *E. coli* by Compact Dry was higher than the 11% detection from the Ojal, while the 5.2% detection of E. coli at concentrations ≥10 *E. coli* per 100cm^2 was comparable to the 5.1% detection of ≥1 *E. coli* per 10 cm^2 from the Ojal kit.
