# Supplementary material for: A localized sanitation status index as a proxy for fecal contamination in urban Maputo, Mozambique
Source: PLoS One. 2019 Oct 25;14(10):e0224333. doi: 10.1371/journal.pone.0224333 (PMC6814227; doi:10.1371/journal.pone.0224333)
Supplement: S3 Fig — (PDF) [file pone.0224333.s008.pdf]

S3 Fig. LSSI Model Diagnostics.

Plotting model residuals (of log_{10} E. coli per gram dry soil) vs. LSSI shows that the linearity assumption in regression is true and the QQ plot shows the residuals are normally distributed. We performed Levene's test on our model, rejected the null hypothesis (p=0.79), and assumed homoscedasticity.

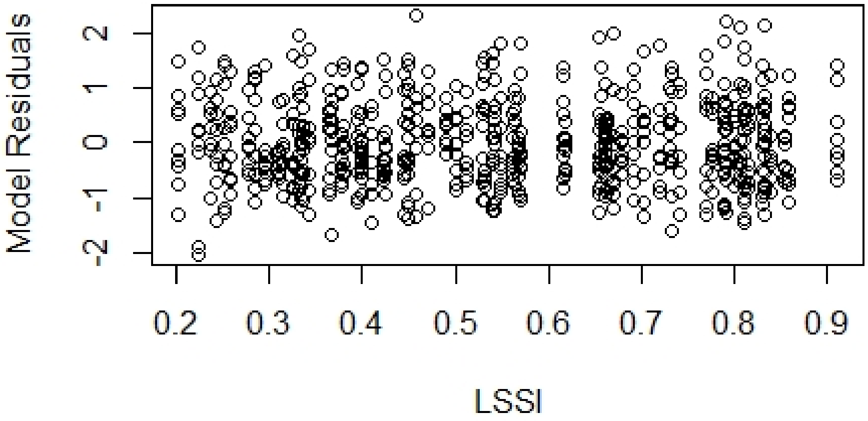

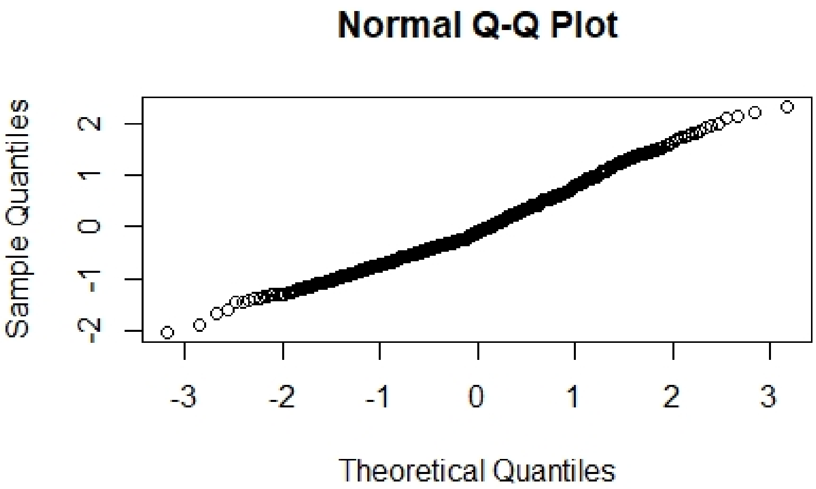

Lmer model output

```
Linear mixed model fit by REML ['lmerMod']
Formula: ecoli_log50 ~ Sun + Local + cssi_10 + Chicken + Duck + poverty_q +
    (1 | compound/household)
   Data: soildata
```

```
REML criterion at convergence: 1792.6

Scaled residuals:
    Min      1Q  Median      3Q     Max
-2.49     -0.65   -0.12    0.64    2.85

Random effects:
 Groups              Name        Variance Std.Dev.
 household:compound  (Intercept) 0.05     0.21
 compound            (Intercept) 0.16     0.40
 Residual                        0.66     0.81
Number of obs: 675, groups:  household:compound, 600; compound, 75

Fixed effects:
             Estimate Std. Error t value
(Intercept)   2.61       0.24      10.76
Sun_partial   0.13       0.12      1.09
Sun_shade     0.39       0.12      3.26
Localfood     0.19       0.14      1.37
Localentrance 0.29       0.14      2.09
LocalHH       0.14       0.14      1.04
LocalHH2      0.14       0.14      0.98
LocalLatrine  0.45       0.14      3.23
Localgarbage  0.74       0.14      5.29
Localdishes   0.82       0.14      5.88
Localclothes  0.75       0.14      5.41
lssi_10      -0.05       0.03     -1.77
ChickenYes    0.65       0.17      3.79
DuckYes       0.42       0.25      1.69
poverty_q    -0.09       0.05     -1.81
```

```
                   2.5 %      97.5 %
.sig01         0.00        0.47
.sig02         0.30        0.49
.sigma         0.70        0.88
(Intercept)    2.14        3.07
Sun_partial   -0.10        0.35
Sun_shade      0.16        0.62
Localfood     -0.08        0.47
Localentrance  0.02        0.56
LocalHH       -0.13        0.42
LocalHH2      -0.13        0.41
LocalLatrine   0.18        0.73
Localgarbage   0.47        1.01
Localdishes    0.55        1.10
Localclothes   0.48        1.02
cssi_10       -0.11        0.00
ChickenYes     0.33        0.99
DuckYes       -0.06        0.90
poverty_q     -0.19        0.01
```

```
anova(Levene.Model) #displays the results
Analysis of Variance Table

Response: Model.Res2
           Df Sum Sq Mean Sq F value Pr(>F)
cssi_10     1   0.04 0.04      0.07   0.79
Residuals 673 391.70 0.58
```
