# Supplementary material for: A localized sanitation status index as a proxy for fecal contamination in urban Maputo, Mozambique
Source: PLoS One. 2019 Oct 25;14(10):e0224333. doi: 10.1371/journal.pone.0224333 (PMC6814227; doi:10.1371/journal.pone.0224333)
Supplement: S2 Table — (PDF) [file pone.0224333.s010.pdf]

S2 Table. Household and compound characteristics.

Category	Description	Data
Respondent	Median time respondents lived in their homes	9 years
	Average time respondents lived in their homes	14 years
Compounds	Average number of families per compound	4
	Average number of people per compound	17
	Average number of children under 5 per compound	2
	Average poverty score (Mozambique Simple Poverty Scorecard)	33/81
Observations	Human feces on the ground or inside the sanitation system	11% (9)
	Used diapers on the ground or in a garbage pile	13% (10)
	Standing water	49% (39)
	Any animal present	59% (47)
	Cat present	40% (32)
	Chicken present	15% (12)
	Ducks present	10% (8)
	Dog present	9% (7)
	Pidgeon(s) present	1% (1)
On-site sanitation	Pour-flush to pit or septic tank	63% (50)
	Pit latrine with concrete slab	16% (13)
	Pit latrine without concrete slab	21% (17)

Note: All compounds contained a second household not enrolled in the MapSan trial that was suitable for environmental sampling. However, at each compound we attempted to interview a third individual, the compound head, in addition to the two households. The data collected from the compound heads was for a separate analysis. Survey questions asked to compound heads were different than the household survey questions. At 13 compounds the second household was the household of the compound head and we were therefore unable to calculate an LSSI for these households.
