# Supplementary material for: A localized sanitation status index as a proxy for fecal contamination in urban Maputo, Mozambique
Source: PLoS One. 2019 Oct 25;14(10):e0224333. doi: 10.1371/journal.pone.0224333 (PMC6814227; doi:10.1371/journal.pone.0224333)
Supplement: S5 Text — (PDF) [file pone.0224333.s011.pdf]

S5 Text. Explanation of soil replicate results

The large average difference between replicate soil samples was skewed from modest variation in highly concentrated samples; 86% of the total variation between replicates came from the 12% of samples with the highest concentrations of *E. coli*. For example, soil sample X with replicate concentrations of 100 and 200 CFU *E. Coli* per gram dry soil and replicate Y with replicate concentrations of 100,000 and 200,000 CFU *E. Coli* per gram dry soil are similarly correlated but the mean difference in replicate values is skewed by the highly concentrated replicate Y. Our protocol diluted soil samples 1:100, therefore the median difference between replicates (2.50 $\log_{10}$ CFU) corresponds to a 3 colony difference between replicate samples. Additionally, the Pearson's correlations coefficient (0.84) suggests a strong positive correlation between replicate soil samples. Given the small median variation between replicates and the strong correlation between replicates, the average of the two replicates from each location is suitable to model *E. coli* in this study.
