# Supplementary material for: A localized sanitation status index as a proxy for fecal contamination in urban Maputo, Mozambique
Source: PLoS One. 2019 Oct 25;14(10):e0224333. doi: 10.1371/journal.pone.0224333 (PMC6814227; doi:10.1371/journal.pone.0224333)
Supplement: S3 Table — (PDF) [file pone.0224333.s012.pdf]

S3 Table. Visibly wet soil by intra-compound location

Intra-compound location	Visibly wet soil
Dishwashing area	90% (n=72/80)
Clothes washing area	85% (n=68/80)
Latrine entrance	69% (n=55/80)
Garbage storage	68% (n=54/80)
Food prep	61% (n=49/80)
MapSan Household	60% (n=48/80)
Compound entrance	54% (n=43/80)
Non-MapSan Household	54% (n=43/80)
Center of the yard	45% (n=36/80)
