# Supplementary material for: A localized sanitation status index as a proxy for fecal contamination in urban Maputo, Mozambique
Source: PLoS One. 2019 Oct 25;14(10):e0224333. doi: 10.1371/journal.pone.0224333 (PMC6814227; doi:10.1371/journal.pone.0224333)
Supplement: S4 Table — (PDF) [file pone.0224333.s013.pdf]

S4 Table. Soil sun exposure by intra-compound location

Intra-compound location	Full Sun	Partial Sun	Full Shade
Center of the yard	29% (n=23/80)	49% (n=39/80)	22% (n=18/80)
Clothes washing area	15% (n=12/80)	40% (n=32/80)	45% (n=36/80)
Food prep	13% (n=10/80)	23% (n=18/80)	65% (n=52/80)
Non-MapSan Household	13% (n=10/80)	40% (n=32/80)	48% (n=38/80)
Compound entrance	11% (n=9/80)	53% (n=42/80)	36% (n=29/80)
Garbage storage	11% (n=9/80)	39% (n=31/80)	50% (n=40/80)
MapSan Household	11% (n=9/80)	39% (n=31/80)	50% (n=40/80)
Dishwashing area	9% (n=7/80)	38% (n=30/80)	54% (n=43/80)
Latrine entrance	8% (n=6/80)	41% (n=33/80)	51% (n=41/80)
