# Supplementary material for: A localized sanitation status index as a proxy for fecal contamination in urban Maputo, Mozambique
Source: PLoS One. 2019 Oct 25;14(10):e0224333. doi: 10.1371/journal.pone.0224333 (PMC6814227; doi:10.1371/journal.pone.0224333)
Supplement: S5 Table — (PDF) [file pone.0224333.s015.pdf]

S5 Table. Association of LSSI variables with *E. coli* counts in soil

		Univariable		Multivariable	
**Indicator**	**Reference**	**Beta**	**95% CI**	**Beta**	**95% CI**
Access to infrastructure	Ten percentage point increase	**-0.09**	**(-0.17, -0.01)**	**-0.07**	**(-0.13, -0.00)**
Containment safety		-0.05	(-0.11, 0.01)	-0.02	(-0.07, 0.03)
Hygiene		-0.04	(-0.09, 0.02)	-0.03	(-0.07, 0.01)
Access to emptying services		-0.02	(-0.05, 0.01)	-0.01	(-0.04, 0.01)
Transport safety		-0.01	(-0.05, 0.03)	-0.01	(-0.04, 0.03)
Final disposal		**-0.04**	**(-0.08, 0.00)**	**-0.04**	**(-0.08, -0.01)**
Access to water supply		-0.01	(-0.04, 0.02)	-0.01	(-0.04, 0.02)
Solid waste management		**-0.07**	**(-0.13 0.00)**	-0.03	(-0.09, 0.03)
Storm- and greywater management		-0.05	(-0.11, 0.01)	-0.03	(-0.08, 0.01)
**Variable**					
Type of on-site sanitation system	Ten percentage point Increase	-0.05	(-0.12, 0.02)	-0.02	(-0.08, 0.03)
Toilet sharing		-0.06	**(-0.11, -0.01)**	**-0.06**	**(-0.10, -0.02)**
Structural stability		-0.02	(-0.06, 0.02)	-0.01	(-0.05, 0.02)
Type of lining		-0.02	(-0.07, 0.02)	-0.01	(-0.05, 0.04)
Superstructure roof		-0.01	(-0.04, 0.02)	-0.01	(-0.03, 0.02)
Superstructure walls		-0.04	(-0.11, 0.03)	-0.03	(-0.09, 0.03)
Containment effectiveness		0	(-0.06, 0.07)	0.03	(-0.02, 0.09)
Groundwater level		-0.03	(-0.07, 0.01)	**-0.03**	**(-0.06, 0.00)**
Hygienic condition		-0.02	(-0.06, 0.01)	-0.02	(-0.05, 0.01)
Soap and water for handwashing		-0.02	(-0.06, 0.02)	-0.01	(-0.05, 0.03)
Type of on-site sanitation lid		-0.01	(-0.05, 0.03)	-0.01	(-0.04, 0.02)
Type of emptying equipment		-0.02	(-0.05, 0.01)	-0.02	(-0.04, 0.01)
Local fecal waste transport		-0.02	(-0.05, 0.02)	-0.01	(-0.04, 0.02)
Neighborhood fecal waste transport		-0.01	(-0.06, 0.05)	0.00	(-0.50, 0.50)
Disposal management		**-0.05**	**(-0.10, 0.00)**	**-0.05**	**(-0.09, -0.01)**
Access to water		-0.01	(-0.04, 0.02)	-0.01	(-0.04, 0.02)
Local solid waste		-0.03	(-0.09, 0.02)	-0.01	(-0.06, 0.04)
Neighbor solid waste		-0.04	(-0.08, 0.01)	**-0.03**	**(-0.06, 0.00)**
Greywater management		-0.02	(-0.07, 0.03)	-0.02	(-0.06, 0.02)
Neighbor stormwater accumulation		-0.03	(-0.7, 0.01)	-0.02	(-0.05, 0.02)
